# Collagen-rich airway smooth muscle cells are a metastatic niche for tumor colonization in the lung

**DOI:** 10.1038/s41467-019-09878-4

**Published:** 2019-05-13

**Authors:** Yu-Cheng Lee, Antonina V. Kurtova, Jing Xiao, Fotis Nikolos, Kazukuni Hayashi, Zoe Tramel, Antrix Jain, Fengju Chen, Mithil Chokshi, Ciaran Lee, Gang Bao, Xiang Zhang, Jianjun Shen, Qianxing Mo, Sung Yun Jung, David Rowley, Keith Syson Chan

**Affiliations:** 1 Department of Molecular and Cellular Biology, Baylor College of Medicine, Houston, TX 77030 USA; 2 Graduate Program in Translational Biology and Molecular Medicine, Baylor College of Medicine, Houston, TX 77030 USA; 3 Department of Biochemistry and Molecular Biology, Mass Spectrometry Proteomics Core, Baylor College of Medicine, Houston, TX 77030 USA; 4 Dan L Duncan Cancer Center, Baylor College of Medicine, Houston, TX 77030 USA; 5 Department of Bioengineering, Rice University Houston, Houston, TX 77030 USA; 6 University of Texas M.D. Anderson Cancer Center, Houston, TX 77030 USA; 7 Department of Biostatistics & Bioinformatics, H. Lee Moffitt Cancer Center & Research Institute, Tampa, FL 33612 USA

**Keywords:** Cancer microenvironment, Cancer stem cells, Metastasis, Preclinical research, Bladder cancer

## Abstract

Metastases account for the majority of cancer deaths. While certain steps of the metastatic cascade are well characterized, identification of targets to block this process remains a challenge. Host factors determining metastatic colonization to secondary organs are particularly important for exploration, as those might be shared among different cancer types. Here, we showed that bladder tumor cells expressing the collagen receptor, CD167a, responded to collagen I stimulation at the primary tumor to promote local invasion and utilized the same receptor to preferentially colonize at airway smooth muscle cells (ASMCs)—a rich source of collagen III in lung. Morphologically, COL3-CD167a-driven metastatic foci are uniquely distinct from typical lung alveolar metastatic lesions and exhibited activation of the CD167a-HSP90-Stat3 axis. Importantly, metastatic lung colonization could be abrogated using an investigational drug that attenuates Stat3 activity, implicating this seed-and-soil interaction as a therapeutic target for eliminating lung metastasis.

## Abstract (web summary)

Collagen is a dynamic component of both the tumor and metastatic niche. Here, the authors show that airway smooth muscle cells are a collagen III rich niche bladder cancer cells expressing CD167a, and Stat3 is a downstream target for abrogating  these collagen III/CD167a-driven metastatic foci.

## Introduction

Metastasis is a multi-step process that includes, but is not limited to local invasion, intravasation of tumor cells into the circulation and extravasation of circulating tumor cells (CTCs) from the circulation into distal organs for metastatic colonization^1–6^. The 5-year survival rate of patients diagnosed with metastatic bladder urothelial carcinoma is ~6%, posing a major clinical challenge^7–10^. Previous studies in human bladder urothelial carcinomas have elegantly investigated the mechanistic contribution of tumor cell intrinsic properties that promote metastasis^11–13^. However, the contribution of tumor microenvironments at both the primary tumor and metastatic sites to the metastatic process is not as well characterized^14^.

Historically, the extracellular matrix (ECM) was considered a passive scaffold, enclosing neighboring cancer cells and other cell types to support tissue architecture. Intriguingly, recent studies revealed the emerging roles of ECMs as a dynamic component of the tumor microenvironment, which crosstalks with cancer cells at the primary tumor site^15–17^. Collagens are major components of the ECM in various organs^15^, including the bladder. Albeit the interaction of collagens with its receptors to facilitate the metastatic cascade, has not been sufficiently explored in bladder carcinomas. Extensive studies have investigated how primary tumors create a favorable microenvironment at secondary organs—designated as the pre-metastatic niche^18^. However, the pre-existence of a physical metastatic niche within secondary organs (e.g. lung) to support Paget’s original seed and soil hypothesis for metastatic seeding and colonization currently lacks evidential substantiality. Here, we report a collagen receptor (CD167a or Discoidin domain receptor 1) on tumor cells, which crosstalk with collagens at both the primary tumor microenvironment and a newly identified metastatic niche in the lung.

## Results

### Collagen I positively correlates with bladder tumor staging

**Fig. 1:**
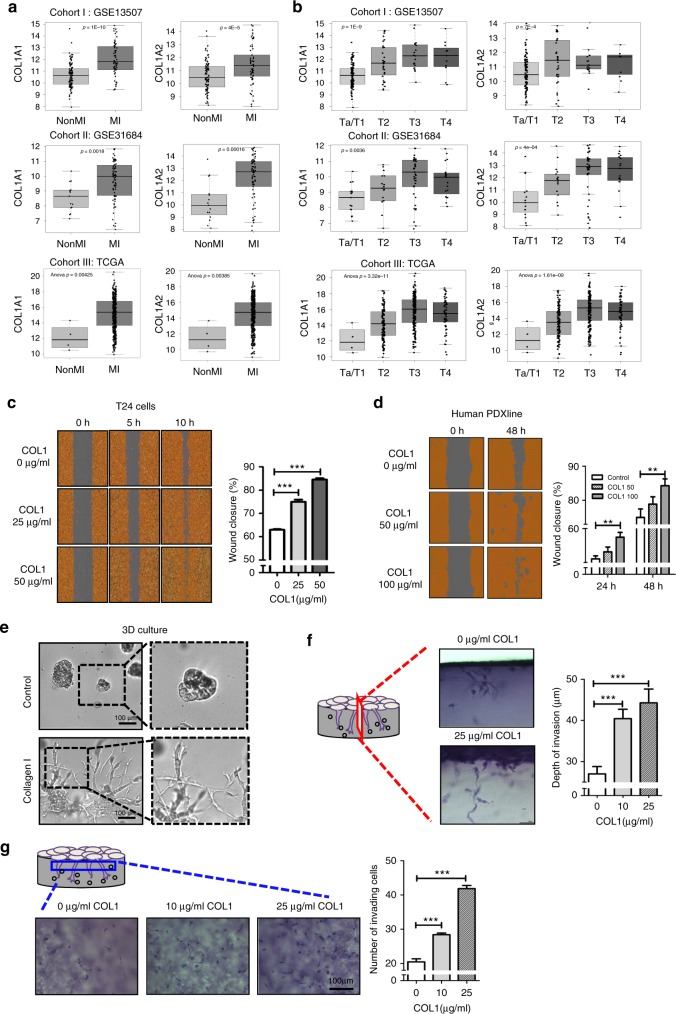
Collagen I expression correlates with increasing tumor stage and functionally enhances local invasion. **a** Comparative analysis of collagen I gene expression (*COL1A1* and *COL1A2*) with non-muscle invasive and muscle-invasive bladder cancer status in three different human bladder cancer patient cohorts (cohort I: kim, *n* = 165^20^, cohort II: Riester, *n* = 93^21^, cohort III: TCGA *n* = 376^22^). **b** Correlation of *COL1A1* and *COL1A2* genes expression with clinical tumor staging (Bladder cancer T staging: pathological evaluation of invasion) in cohorts from **a**. **c** Dose-dependent treatment of exogenous collagen I (0, 25, and 50 μg ml^−1^) and its effects on T24 cell migration. Left panel: Representative images of wound closure at 0, 5, and 10 h under collagen I treatment. Right panel: Quantification of collagen I-induced percent migration at 10 h post-wound induction relative to 0 h. **d** Dose-dependent treatment of collagen I (50 and 100 μg ml^−1^) and its effects on a patient-derived xenograft (PDX) culture cell migration. Left panel: Representative images of wound closure at 0 and 48 h of collagen I treatment. Right panel: Quantification of collagen I-induced percent migration at 24 and 48 h post-wound induction relative to 0 h. **e** Representative images of T24 cancer cells cultured in a three-dimensional (3D) matrigel matrix in the absence (top panel, control) or presence (bottom panel) of collagen I (0.25 mg ml^−1^). **f**, **g** The 3D-invasive capacity of T24 cells in the presence or absence of collagen I treatment (0, 10 or 25 μg ml^−1^) for 48 h. Representative photos of perpendicular (**f**, left panel) and horizontal sections (**g**, left panel) of tumor cells invading through the matrix. The distance and the corresponding number of invading cells from the monolayer into the matrix were quantified as presented in right panel of graft **f**, **g**, respectively. Statistical analysis: **a**, **b** Analysis of Variance test (ANOVA); **c**–**g**, a two-tailed, unpaired student’s *t*-test. Error bar: mean ± SEM, *n* = 3 independent experiments. ^**^p < 0.01, ^***^p < 0.005

Clinically, tumor (T) staging defines the invasiveness of human bladder urothelial carcinoma cells into smooth muscle and perivesical tissues pathologically^19^, with Ta/T1 defining non-muscle invasive bladder carcinomas (NMIBCs), and T2-T4 representing muscle-invasive bladder carcinomas (MIBCs). To explore a clinical connection between collagen I (COL1) and tumor cell invasion, we evaluated collagen I gene expression (*COL1A1* and *COL1A2*) in correlation to tumor staging in three independent cohorts of bladder cancer patients [kim, *n* = 165^20^, Riester, *n* = 93^21^ and The Cancer Genome Atlas (TCGA), *n* = 376^22^] (Fig. 1a, b). Initial analysis of *COL1A1* and *COL1A2* genes revealed significantly higher expression in MIBCs (>T2), when compared to NMIBCs (Ta/T1) [Fig. 1a, cohort I: *COL1A1 p* = 1E-10; *COL1A2 p* = 4E-5; cohort II: *COL1A1 p* = 0.0018; *COL1A2 p* = 0.00016; cohort III: *COL1A1 p* = 0.00425; *COL1A2* p = 0.00385]. Subsequent detailed analysis also revealed a significant and positive correlation between *COL1A1* and *COL1A2* gene expression with increasing tumor staging [Fig. 1b, cohort I: *COL1A1 p* = 1E-9 and *COL1A2 p* = 7E-4, Ta/T1 (*n* = 103), T2 (*n* = 26), T3 (*n* = 13), T4 (*n* = 23); cohort II: *COL1A1 p* = 0.0036 and *COL1A2 p* = 4E-04, Ta/T1 (*n* = 15), T2 (*n* = 17), T3 (*n* = 42), T4 (n = 19); cohort III: *COL1A1 p* = 3.32E-11, and *COL1A2* *p* = 1.61E-09, Ta/T1 (*n* = 4), T2 (*n* = 120), T3 (*n* = 194), T4 (*n* = 58)]. Ta/T1 tumors are papillary and non-muscle invasive carcinomas, respectively. T2 tumors represent carcinomas beginning to invade into the smooth muscles, T3 tumors represent those that have invaded through muscularis into perivesical adipose tissues, and T4 tumors have spread beyond perivascular adipose tissues into nearby organs. Since collagens constitute the major proteins comprising the ECM, and were also recognized as functional ligands that facilitate crosstalk with neighboring cancer cells^15–17,23^, the findings in Fig. 1a, b implicated the clinical relevance to further investigate collagen I and its crosstalk with collagen receptor on cancer cells to regulate tumor invasion.

### Collagen I enhances tumor cell migration and invasion

To evaluate COL1 as a functional ligand to modulate the invasive properties of human bladder cancer cells, we utilized a weakly metastatic cancer cell line T24^24^, as well as primary cultures from a metastatic patient-derived xenograft (Human PDX) to assess their responses to exogenous COL1 treatment in vitro. First, to examine whether COL1 regulates cell migration, cancer cells were subjected to an in vitro scratch assay^25^, in the absence or presence of increasing exogenous COL1 (25, 50, and 100 μg ml^−1^) (Fig. 1c, d). COL1 treatment consistently enhanced wound closure in a dose-dependent manner, in both T24 and human PDX models, when compared to vehicle control (Fig. 1c, d); thus, confirming COL1 is not merely a passive ECM, but also a functional ligand that promotes cell migration.

Next, to explore the functional impact of COL1 in modulating cancer cell invasion through ECM, we employed various 3-dimensional (3D) models to better recapitulate in vivo conditions. When cancer cells were cultured at low density within a thick layer of matrigel matrix (Fig. 1e, upper panels) or collagen-matrigel mixed matrix (Fig. 1e, lower panels), they exhibited significantly distinct cellular morphologies. Cancer cells embedded within the collagen-matrigel matrix exhibited extensive sprouting structures with a higher propensity to invade through the COL1-enriched matrix (Fig. 1e, lower panels), while cells embedded within matrigel remained primarily spherical (Fig. 1e, upper panels). Collectively, extracellular COL1 consistently increases cell migration in 2D; however, its functional impact on cancer cells is much more pronounced within a 3D matrix. To quantify the capacity of COL1 to stimulate invasion through 3D matrice, we employed another model comprising two layers of matrices^26^ (Fig. 1f, g). This 3D invasion assay revealed that COL1 was sufficient to stimulate a longer invading distance by cancer cells into the bottom matrix (Fig. 1f), as well as increased the total number of cells that invaded into the matrix in a dose-dependent manner (Fig. 1g). Collectively, these results convincingly demonstrated collagen I is not only a passive ECM component, but also a functional ligand that promoted cancer cell sprouting and invasion through ECM.

### CD167a as a collagen receptor in human MIBCs

**Fig. 2:**
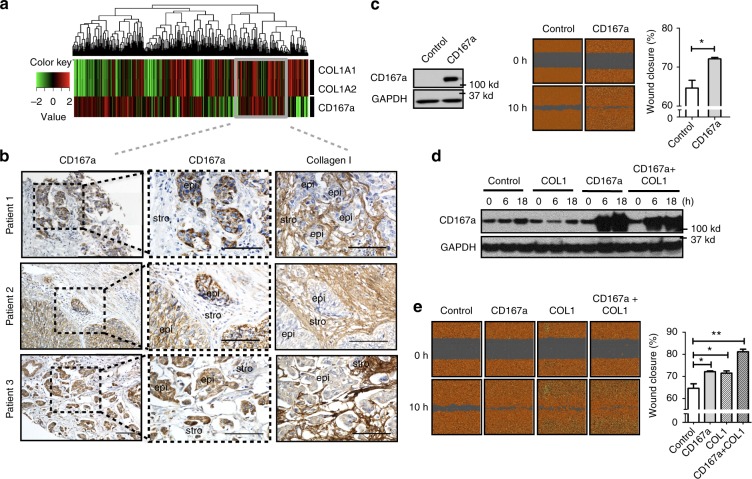
CD167a expression enhances cell migration in vitro. **a** Hierarchical clustering of *COL1A1, COL1A2* (collagen genes), and *CD167a* (collagen receptor) genes expression in a human bladder cancer patient cohort (cohort III: TCGA); red and green colors indicate high and low expression, respectively. Grey box indicates patients with co-expression of *COL1A1/COL1A2* and *CD167a* genes. **b** Immunohistochemical analyses of collagen I and CD167a in representative human MIBC tissues verified the localization of CD167a positive cancer cells in adjacent to stromal collagen I expression. Scale bar:100 μm. **c** Left panel: Western blot analyzing CD167a protein expression in mCherry-CBLuc-Control and mCherry-CBLuc-CD167a-T24 cancer cells. Middle panel: Representative images of mCherry-CBLuc-Control and mCherry-CBLuc-CD167a-T24 cancer cell migration capacity in vitro. Right panel: Quantification of percent migration at 10 h post-wound induction relative to 0 h. **d**, **e** Combinatorial effects of exogenous collagen I and CD167a overexpression in cancer cell migration in vitro. Doxycycline-inducible CD167a-expressing T24 cancer cells were subjected to the wound-healing assay with or without collagen I treatment. Cell lysates were harvested after collagen I and doxycycline (15 ng/ml) stimulation for subsequent western blot evaluation at the indicated time points (0, 6, and 18 h). Left panel: Representative images of wound closure at 0 and 10 h upon treatment with 25 μg ml^−1^ collagen I. Right panel: Quantification of percent migration at 10 h post-wound induction relative to 0 h. Statistical analysis: a two-tailed, unpaired student’s *t*-test. Error bar: mean ± SEM, *n* = 3 independent experiments. ^*^p < 0.05, ^**^p < 0.01

To identify the relevant collagen receptor, we analyzed gene expression dataset of human MIBCs and identified a patient population co-expressing both COL1A1/1A2 and the collagen receptor CD167a (or Discoidin domain receptor 1; Fig. 2a, i.e. heatmap), validated at the protein level by immunohistochemical analysis, utilizing tissue sections from human MIBC patients (Fig. 2b). Immunohistochemistry revealed a series of human MIBCs (>T2) expressing COL1 in the surrounding stromal regions, with neighboring cancer cells staining positive for cell surface CD167a (Fig. 2b). Additionally, we found that exogenous COL1 treatment of T24 bladder cancer cells significantly enhanced the mRNA expression of CD167a, but not other candidate collagen receptors, nor to the extent of CD167a induction (Supplementary Fig. 1). These findings led us to further investigate CD167a as a collagen receptor during bladder tumorigenesis.

### COL1-CD167a signaling enhances MIBC migration and invasion

To further evaluate the biological roles of CD167a, we generated a lentiviral construct driving CD167a gene expression and stably transduced this vector into T24 cells (a weakly metastatic bladder cancer cell line). The endogenous protein level of CD167a was relatively low in T24 cells, overexpression of CD167a protein was validated by western blot analysis (Fig. 2c). To assess the migratory and invasive ability of CD167a-overexpressing cancer cells, we subjected them to the 2D cell-scratch assay and the 3D invasion assay. CD167a overexpression significantly promoted the migration of cancer cells to enhance wound closure (Fig. 2c, *p* = 0.028), and increased cancer cell invasion into 3D matrices (Supplementary Fig. 2a–c, *p* < 0.001). These data confirmed the roles of CD167a overexpression in promoting cell migration and local invasion. Further functional studies revealed a cooperative effect of exogenous COL1 and CD167a receptor expression in an augmented migratory capability of CD167a-overexpressing T24 bladder cancer cells to initiate wound closure (Fig. 2d, e). Collectively, these findings established a biological role for COL1 to activate CD167a on cancer cells, enhancing cell migration and local invasion within the tumor microenvironment.

### CD167a promotes distal lung metastasis in vivo

**Fig. 3:**
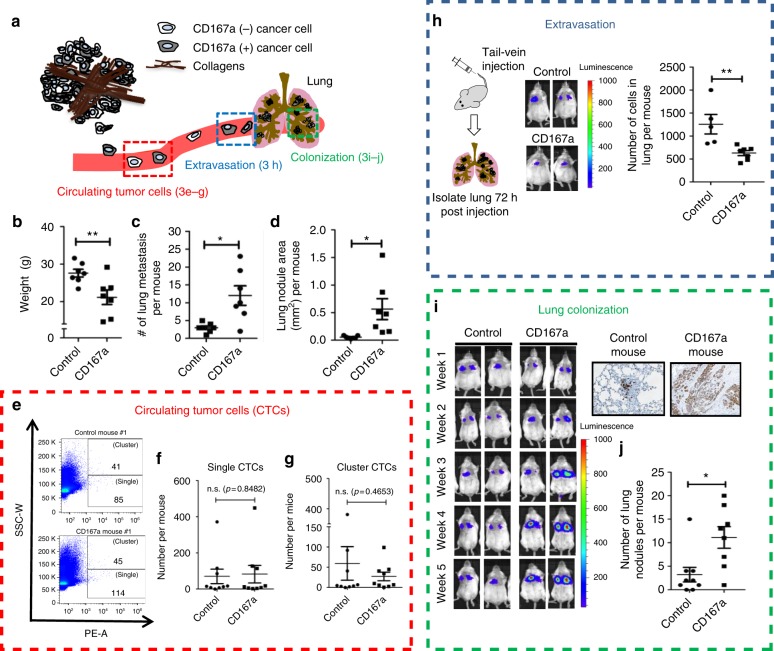
CD167a facilitates lung colonization in vivo. **a** Schematic representation of the metastatic cascade: this includes intravasation of tumor cells into the circulation as circulating tumor cells (CTCs) (red box), extravasation of CTCs from the circulation (blue box), and colonization at distant organ, e.g. lung (green box). **b** Endpoint analysis of mouse weight ectopically injected with control or CD167a-overexpressing T24 cancer cells (*n* = 7) over a 12-week period. **c** Quantification of metastatic nodules in lung of mice injected with control or CD167a-overexpressing T24 cancer cells (*n* = 7). **d** Quantification of metastatic nodule total area (mm^2^) in lungs of mice injected with control or CD167a-overexpressing T24 cancer cells (*n* = 7). **e**–**g** The examination of CTCs frequency in a spontaneous metastatic model. Peripheral blood was collected from mCherry-CBLuc-control or CD167a-overexpressing mCherry-CBLuc-T24 cells tumor-bearing mice and analyzed by flow cytometry to quantify fluorescent CTCs. Frequency of single and cluster CTCs is shown in **f**, **g**, respectively. *n* = 9. **h** Left panel: Schematic illustration of the tail-vein injection model. Middle panel: Representative bioluminescence images (BLI) of mice injected with control or CD167a-overexpressing mCherry-CBLuc-T24 cancer cells. Right panel: Quantification of cancer cell extravasation in the lungs of mice injected with control (*n* = 5) or CD167a-overexpressing mCherry-CBLuc-T24 cancer cells (*n* = 7) 72 h post-injection after immunohistochemical analysis. **i** Left panel: Representative bioluminescence images of mice injected with mCherry-CBLuc-control or mCherry-CBLuc-CD167a-overexpressing-T24 cancer cells via tail-vein, temporally tracked for tumor colonization up to 5 weeks post injection (Control *n* = 9, CD167a, *n* = 8). **j** Histological lung sections from mice injected with control and CD167a cancer cells from **i** were independently analyzed for graphical quantification of metastatic nodules in the lung. Statistical analysis: a two-tailed, unpaired Student’s *t*-test. Error bar: mean ± SEM. ^*^p < 0.05, ^**^p < 0.01

To further evaluate the in vivo functional roles of CD167a within the metastatic cascade, we engineered the weakly metastatic T24 cancer cells to carry mCherry fluorescence and click-beetle luciferase (CBLuc) reporters for in vivo tracking. We then compared T24 cancer cells overexpressing CD167a to parental cells in their ability to initiate distal lung metastases (Fig. 3a). First, we utilized a spontaneous metastasis model by ectopically inoculating 1 × 10^6^ CD167a-overexpressing or empty-vector transduced T24 cancer cells into Rag2^−/−^; IL-2Rγc^−/−^ immunocompromised mice, and monitored secondary organ metastasis over time using IVIS imaging. At ~4–6 weeks post tumor inoculation, mice harboring CD167a-overexpressing xenograft tumors started exhibiting a notable loss in body weight when compared to vector controls, which became statistically significant at 10–12 weeks (Fig. 3b, control *n* = 7, CD167a *n* = 7). Since one of the clinical symptoms of metastatic cancer patients is unintended weight loss, and taking into consideration that T24 cancer cells could form lung metastasis^24^, we speculated the unintended weight loss observed in the CD167a-overexpressing group likely resulted from accelerated distal metastases. We therefore analyzed the gross morphology and histopathology of lung tissues collected from both experimental groups (Fig. 3c, d, control *n* = 7, CD167a *n* = 7**)**. At the time of sacrifice, histologic analysis by H&E staining uncovered microscopic metastases in both groups; interestingly, there was a statistically significant increase in the number (Fig. 3c) and size (Fig. 3d) of metastatic lung nodules formed from CD167a-overexpressing T24 cells, when compared with parental cells in this spontaneous metastatic model. Collectively, these findings revealed a role for CD167a in enhancing the development of distal lung metastases in an human bladder cancer model in vivo.

### CD167a does not impact tumor cell intravasation

To mechanistically dissect the roles of CD167a during the metastatic cascade (Fig. 3a), we first evaluated its capacity to facilitate tumor intravasation into the circulation as CTCs (Fig. 3e). The analyses of CTCs were performed using the spontaneous metastatic model, comparing mice ectopically injected with CD167a-overexpressing (*n* = 9) or vector transduced T24 cells (n = 9). After ~8–9 weeks post tumor inoculation when tumor volume reached approximately 100 mm^3^, peripheral blood was collected and subjected to flow cytometric analysis (Fig. 3e). Since we engineered these cancer cells to carry the fluorescent protein mCherry, we could readily detect CTCs in peripheral blood via their fluorescent intensity using flow cytometry (Supplementary Fig. 3). Interestingly, there were no statistically significant difference in the number of CTCs detected as single cells between the two groups (Fig. 3f; Control group = 0–371 CTCs, CD167a group = 3–448 CTCs). A recent report illustrated CTC clusters possess greater metastatic potential than single CTCs^27^; therefore, we further evaluated the occurrence of CTC clusters. Nonetheless, the frequency of CTC clusters between the two groups was not significantly altered (Fig. 3g; Control group = 0–381 CTCs, CD167a group = 1–99 CTCs). Together, these findings revealed tumor intravasation into the circulation was unlikely a primary mechanism for CD167a to enhance distal metastasis.

### CD167a does not enhance extravasation to mediate metastasis

Next, to address whether CD167a played a role in regulating extravasation from the circulation into lung, we employed a standard tail-vein injection model (Fig. 3h)^28^. The tail-vein injection assay is a relevant experimental metastasis model to investigate extravasation, since blood vessels in lung are the first capillary bed encountered by cancer cells after injection. Briefly, 1 × 10^6^ CD167a-overexpressing or vector transduced T24 cells were injected into the tail-vein, allowing temporal tracking of extravasation into lung tissues (Supplementary Fig. 4a). The efficiency of tumor cell extravasation into lung tissues was quantified at 72 h post injection (control *n* = 5, CD167a *n* = 7), via bioluminescence imaging (BLI) (Fig. 3h), and IHC staining (Supplementary Fig. 4b). Intriguingly, while CD167a-expressing cancer cells were more efficient in forming distal lung metastases, they were not as efficient in extravasating into lung tissues at this early time point (Fig. 3h), revealing extravasation was not a major mechanism responsible for their enhanced metastasis.

### CD167a enhances metastatic lung colonization

To further evaluate metastatic lung colonization in vivo, the tail-vein metastasis assay was repeated (Fig. 3j, and Supplementary Fig. 4a), and bioluminescent activity was longitudinally tracked by IVIS imaging weekly to monitor lung colonization over a 5 week period. While both groups exhibited a temporal increase in bioluminescent signal (Fig. 3i, control *n* = 9, CD167a *n* = 8), a notable increase of signal intensity in the CD167a-overexpressing group was observed as early as 3 weeks post injection, which became statistically significant at week 5 during the time of sacrifice (Fig. 3i). Detailed histologic analysis confirmed a significant increase in the number of metastatic foci in mice inoculated with CD167a-overexpressing bladder cancer cells, when compared with empty vector transduced group (Fig. 3j); thus, validating a role for CD167a in enhancing metastatic colonization in lung.

### Airway smooth muscle cells as a metastatic niche in lung

**Fig. 4:**
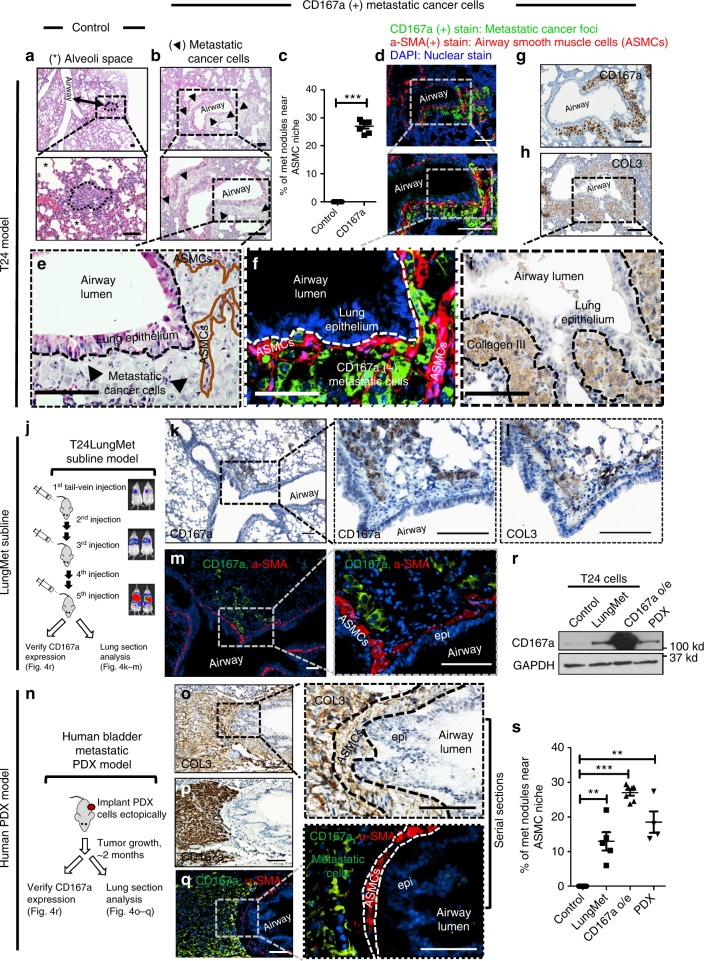
Airway smooth muscle cell (ASMC) is a metastatic niche in the lung. **a** H&E staining of lung sections revealing typical metastatic lung foci within alveolar spaces that were formed from vector control T24 cancer cells. (*) denotes alveolar spaces. Scale bar:100 μm. **b** H&E staining of lung sections revealing morphologically distinct metastatic foci residing adjacent to ASMCs (outlined by arrows), which formed from CD167a-overexpressing T24 cancer cells. Scale bar:100 μm. **c** Quantification of unique lung metastases with proximal ASMCs localization (control, *n* = 7; CD167a, *n* = 7). **d** Immunofluorescent co-staining of α-SMA (an ASMCs marker, red), and CD167a (to label metastatic cancer cells, green), in a serial section to **b**. Scale bar:100 μm. **e**–**i** Serial sections demonstrating H&E staining, IF and IHC staining of various markers illustrating the colonization of CD167a-expressing cancer cells within COL3-rich ASMCs. Anti-CD167a antibody was used to outline metastatic cancer cells, anti-α-SMA and anti-collagen III antibodies were used to label ASMCs and secreted collagens, respectively. Scale bar:100 μm. **j** Serial transplantation scheme to establish a highly-metastatic model (T24-LungMET) that efficiently colonized into lung tissues, by serially transplanting parental T24 cells into tail vein five consecutive times, monitored by bioluminescence imaging. **k**, **l** Representative serial sections of metastatic foci stained with anti-CD167a to mark metastatic cancer cells and its relative localization to COL3 in T24-LungMET model. **m** Immunofluorescence co-staining images of CD167a (green) and α-SMA (red) showing the relative localization of CD167a+ metastatic cancer cells and their proximity to α-SMA+ ASMCs. Scale bar:100 μm. **n** Experimental scheme to establish a spontaneous human bladder metastatic PDX model. **o**, **p** Representative serial sections of metastatic foci stained with anti-CD167a to mark metastatic cancer cells and their proximity to COL3. **q** Immunofluorescence co-staining images of CD167a (green) and α-SMA (red) showing the relative localization of CD167a+metastatic cancer cells and their proximity to α-SMA+ASMCs. Scale bar:100 μm. **r** Western blot analysis comparing CD167a protein expression in parental T24 cells, T24-LungMET model, CD167a-overexpressing T24 model and the human bladder metastatic PDX model. **s** Quantification of lung metastases proximal to ASMCs, in the metastatic bladder carcinoma models evaluated in **r**. Statistical analysis: a two-tailed, unpaired student’s t-test. Error bar: mean ± SEM. **p < 0.01, ***p < 0.005

Another intriguing finding was uncovered by detailed pathological analysis of corresponding metastatic foci via H&E staining. While empty vector transduced cells formed typical metastatic nodules within alveolar spaces (Fig. 4a, * indicates alveolar space), the morphology and colonization pattern of CD167a-overexpressing metastatic foci were noticeably different (Fig. 4b, arrows outlining the areas of metastatic cancer). A notable fraction of CD167a-expressing metastatic foci uniquely resided along or within airway smooth muscle cells (ASMCs), representing an original and newly-described colonization site for metastatic foci (Fig. 4b). Additional examples of these unique metastatic foci resembling early colonization (Supplementary Fig. 5a, b) or overt lung metastases (Fig. 4b and Supplementary Fig. 5c–i) were presented, with their proximal localization adjacent to, or within ASMCs, respectively. Quantification of these two types of metastatic foci indicated a statistical significance (*p* < 0.0001), with CD167a-expressing foci preferentially localized to ASMCs (Fig. 4c, control *n* = 7, CD167a *n* = 7). To validate their unique localization within ASMCs, an antibody against alpha smooth muscle actin (α-SMA) was used to outline ASMCs by immunofluorescence staining (Fig. 4d; red color, and Supplementary Fig. 5e, f), and anti-CD167a was used to mark the relative localization of metastatic cancer cells (Fig. 4d; green color, and Supplementary Fig. 5e, f). Immunofluorescence co-staining validated the colonization of CD167a-expressing cancer cells (Fig. 4d, green) within α-SMA(+) airway smooth muscle cells (Fig. 4d, red), confirming H&E staining results in serial section (Fig. 4e, f, higher magnification). To our knowledge, ASMCs had not been reported to function as supporting niche cells in cancer-related studies. Independently, ASMCs were reported to mediate ECM remodeling by depositing collagen in asthma research studies^29,30^, where type III collagen (COL3) was reported to be the predominant collagen expressed in lung^31,32^. Since COL3 could also act as a ligand for CD167a^33,34^, it led us to hypothesize that ASMCs might be a source of collagen, thus providing a supportive niche for the colonization of CD167a-expressing cancer cells. To evaluate this, immunohistochemical staining of CD167a and COL3 were performed in serial sections (Fig. 4g, h), revealing metastatic cancer cells residing within ASMCs (Fig. 4g) are abundantly surrounded by COL3 (Fig. 4h, i). Such observations extended to metastatic foci representing early colonization, i.e. small clusters of cancer cells aligning adjacent to COL3-rich ECM (Supplementary Fig. 5 area I:a, b, and area II:c–f), and larger nodules of metastatic cancer masses entirely surrounded by COL3 positive areas (Supplementary Fig. 5g–i, area III).

To establish the generalization of airway smooth muscle cells as a metastatic niche, we evaluated additional models of lung metastases (Fig. 4j–s). These included a selected model of highly metastatic T24 subline (Fig. 4j–m, *n* = 5), and a human patient-derived xenograft that spontaneously metastasized to lung (Fig. 4n–q, *n* = 4). The highly metastatic subline (T24-LungMet) was generated from parental T24 cancer cells by serially re-injecting five consecutive times through tail-vein in vivo (Fig. 4j). IVIS imaging (Fig. 4j) and H&E staining (Supplementary Fig. 6) indicated its enhanced metastatic capacity to colonize lung tissues. Using CD167a staining to outline metastatic cancer cells, and α-SMA to outline ASMCs, we identified prevalent metastatic foci localized within COL3-rich ASMCs from the T24-LungMet model (Fig. 4j–m), as well as the spontaneous lung metastatic model of human PDX (Fig. 4n–q). Further, western blot analysis of CD167a protein level in these three independent lung metastatic models (Fig. 4r) revealed an association to the frequency of metastatic foci to airway smooth muscle cells (Fig. 4s), Taken together, these results illustrated airway smooth muscle cells as a newly-identified metastatic niche within the lung microenvironment.

### ASM cells secret collagen III to enhance colonization

**Fig. 5:**
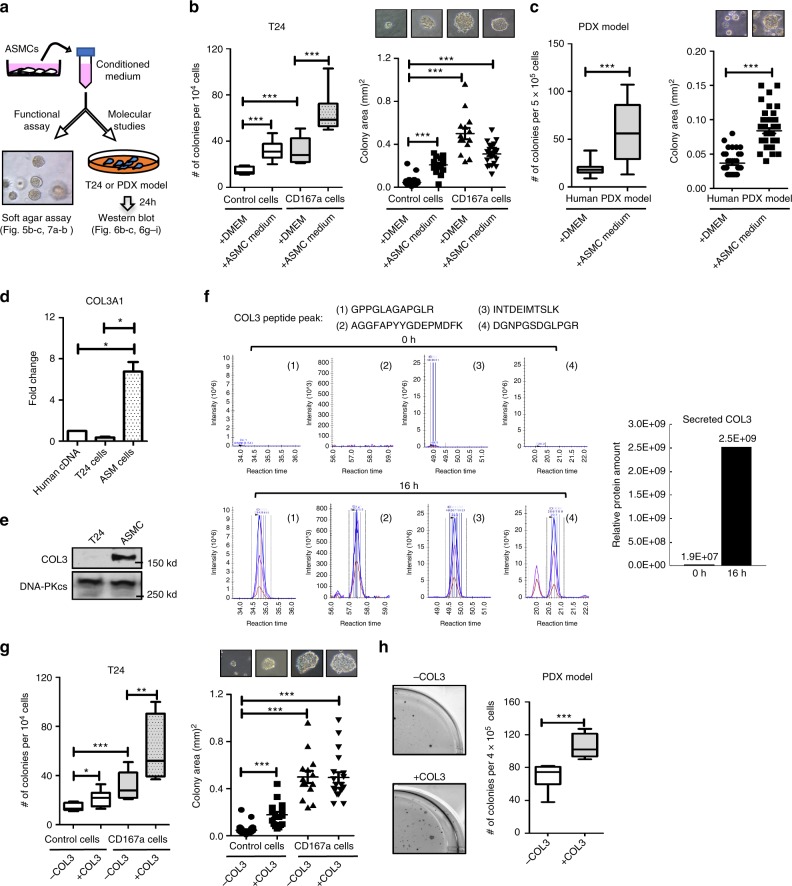
Effects of airway smooth muscle cells and collagen III in colony formation. **a** Schematic illustrating the overall scheme of conditioned media collection from airway smooth muscle cells (ASMCs) for the evaluation of its impact on colony formation in vitro, and subsequent molecular analysis. **b** Quantification and size measurement of colonies formed from control and CD167a-overexpressing T24 cancer cells, and **c** a human metastatic PDX model in a classical soft-agar assay, in the presence or absence of ASMC conditioned media over a 14-day period. Representative phase-contrast images depict the relative colony size from respective conditions. **d** Quantitative real-time qPCR analysis of COL3A1 mRNA in cultured human ASMCs. Human cDNA mix and cultured T24 cells were harvested as controls. GAPDH was used as reference and the relative expression was normalized against human cDNA mix. **e** Western blot analysis of COL3 expression in human ASMCs and T24 cancer cells. DNA-PKcs was used as the loading control. **f** Targeted mass spectrometry analysis to evaluate protein peptides specific toward COL3, that were secreted into the supernatant or conditioned media collected from cultured human ASMCs at 0 h and 16 h after incubation. The amount of COL3 was calculated based on MS1 signal area under curve using SKYLINE software based on top four strongly detected peptides. **g** Colony formation assay of control and CD167a-overexpressing T24 cancer cells upon COL3 treatment (50 μg ml^−1^). Number and size of colonies are shown on the left and right panel, respectively. **h** Colony formation assay of the human metastatic PDX model upon COL3 treatment (50 μg ml^−1^). Statistical analysis: a two-tailed, unpaired student’s *t*-test. Error bar: mean ± SEM. ^*^p < 0.05, ^**^p < 0.01, ^***^p < 0.005

To investigate whether ASMC-secreted host factors could influence colonization in lung, we collected conditioned media from human ASMCs and evaluated its effects in altering the colony-formation capacity of tumor cells, utilizing the soft agar assay (as a surrogate for lung colonization; Fig. 5a). Both the T24 cells and human bladder PDX culture were selected as representative models (Fig. 5b, c). Conditioned media from ASMCs significantly enhanced the frequency of colony formation in parental T24 cells (Fig. 5b, open boxes), and even more so in the CD167a-overexpressing T24 cells (Fig. 5b, grey boxes). A similar finding was observed in the human metastatic PDX model, as ASMC-conditioned media significantly enhanced the frequency and size of colony formation (Fig. 5c). Since our primary emphasis was collagen secretion from ASMCs as the putative ligand for CD167a, and prior studies revealed COL3 as a predominant collagen within lung tissues, we analyzed the relative mRNA and protein expression of COL3 in cultured human ASM cells. COL3 mRNA expression was markedly higher in ASM cells than in a commercially available human cDNA mix or T24 cancer cells (Fig. 5d). Similarly, COL3 protein was abundantly expressed within ASM cells as demonstrated by western blot analysis (Fig. 5e). Additionally, COL3 was detected as secreted protein peptides in the ASMC conditioned media via targeted mass spectrometry (Fig. 5f). Collectively, these results verified COL3 as a secreted protein by ASM cells (Fig. 4f and Supplementary Fig. 7), substantiating the tissue level results from Fig. 4.

To evaluate whether COL3 could recapitulate the effects of ASMC-conditioned media in impacting colony formation, COL3 was embedded in the top layer of the soft agar matrices with tumor cells. Intriguing, COL3 phenocopied the effects of ASMC-conditioned media, which increased colony formation significantly in control and CD167a-overexpressing T24 cancer cells (Fig. 5g), as well as the metastatic PDX (Fig. 5h). Collectively, these results confirmed a role for ASMCs and COL3 in promoting colony formation.

### COL3-CD167a-Stat3 axis in the lung microenvironment

**Fig. 6:**
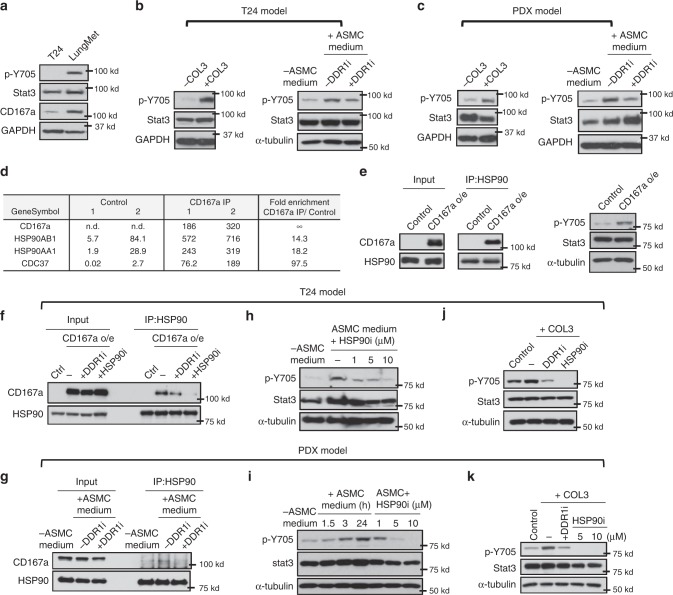
CD167a-Stat3 axis in the COL3-rich airway smooth muscle cell metastatic niche in lung. **a** Western blot evaluating the relative protein expression of CD167a, Stat3 and phosphotyrosine Stat3 (Y705) in parental T24 cells and our newly-established metastatic model (T24-LungMET). **b**, **c** Effects of COL3, and ASMC-conditioned media and CD167a/DDR1 kinase inhibitor (DDR1-IN-1; 10μM) on Stat3 and phosphotyrosine Stat3 protein expression, in T24 and PDX models. **d** Coupling CD167a co-IP with mass spectrometry to discover CD167a-interacting partners. Selected CD167a associated gene protein products (GPs) and their corresponding iBAQ values are listed. **e** Coupling co-IP and western blot to evaluate CD167a-HSP90 interaction, via co-IP using anti-HSP90 antibody followed by western blotting of CD167a in control and CD167a-overexpressing T24 cancer cells. **f** Effects of CD167a kinase inhibitor (DDR1-IN-1, 10μM) and HSP90 inhibitor (Geldanamycin, 5 μM) on CD167a-HSP90 complex formation. Control cells and CD167a-overexpressing T24 cancer cells were treated with COL3 in the absence or presence of indicated inhibitors for 24 h. Cell lysates were collected for co-immunoprecipitation assay using anti-HSP90 antibody. **g** PDX cells were cultured in control (-ASMC) or ASMCs conditioned media (+ASMC) with or without DDR1-IN-1 inhibitor (10 μM) for 24 h. Cell lysates were assessed for CD167a-HSP90 interaction by co-immunoprecipitation assay followed by western blot analysis. **h**, **i** Western blot analysis of Stat3 tyrosine phosphorylation upon treatment with ASMC-conditioned medium in the presence or absence of HSP90 inhibitor (+HSP90i, 1, 5, and 10 μM) in T24 and PDX models. **j**, **k** Western blot analysis of COL3-induced Stat3 tyrosine phosphorylation in T24 and PDX models upon treatment with CD167a/DDR1 kinase specific inhibitor (DDR1-IN-1, 10 μM) and Geldanamycin (HSP90i, 5 μM)

To identify the downstream mediator for the COL3-CD167a axis, we evaluated our newly established metastatic T24-LungMet subline from Fig. 4j. Enhancement of metastatic potential in this T24-LungMet subline associated with a significant increase in CD167a protein expression, which corresponded with an elevated phospho-tyrosine level of Stat3 at a.a. pY705 (Fig. 6a); thus, implicating a molecular link. To evaluate whether ASMCs/COL3 goes through a CD167a-Stat3 axis, we first examined whether ASMC-derived conditioned media or exogenous COL3 treatment had any effects in modulating Stat3 phospho-tyrosine (pY705) level. Both treatment conditions enhanced phospho-tyrosine Stat3 level in the T24 bladder cancer and the human PDX model (Fig. 6b, c), which was significantly diminished by a CD167a/DDR1 selective kinase inhibitor (DDR1-IN-1) (Fig. 6b, c). Since the DDR1-IN-1 compound was originally discovered as a selective kinase inhibitor that blocks CD167a/DDR1 autophosphorylation^35^, these results connected Stat3 as a downstream molecule to the COL3-CD167a canonical axis. Alternatively, a recent report revealed CD167a (or DDR1) can mediate signals via a non-canonical pathway in breast cancer metastases^36^. Our results using the selective kinase inhibitor supported a canonical kinase-dependent role of CD167a/DDR1 in activating Stat3 in our bladder cancer metastasis models (Fig. 6b, c), highlighting the significance of organ-site specificity, postulating discrete canonical and non-canonical CD167a/DDR1 signaling in different tissue types.

To uncover a mechanistic link, we performed a proteomics screen by coupling co-immunoprecipitation with mass spectrometry (IP-MS)^37^ to pull down and categorize CD167a-interacting proteins. Interestingly, HSP90 and CDC37, two proteins forming a chaperone complex, are identified among the top of the list (Fig. 6d). HSP90 functions as a molecular chaperone that modulates its client proteins to impact their protein folding, stability and activation^38^. Hence, HSP90 complexes participate in various biological functions, and signal transduction. Furthermore, it has been reported that Stat3 is also a client protein of HSP90^39,40^, therefore, implicating CD167a/HSP90 as a candidate regulator of Stat3 activation in our cancer model.

To further explore this mechanistic link of CD167a-HSP90-Stat3, we performed reciprocal co-IP using an anti-HSP90 antibody to pull down its interacting protein complexes in control and CD167a-overexpressing T24 cells. Our co-IP results validated the IP-MS profiling results in Fig. 6d, clearly showing protein complex formation between CD167a and HSP90 in our cancer model (Fig. 6e, left panel). Further, Stat3 tyrosine phosphorylation indicative of its activity is increased in CD167a-overexpressing T24 cells (Fig. 6e, right panel), closely associated with an increased complex formation between CD167a and HSP90 (Fig. 6e, left panel). To further evaluate whether this CD167a/HSP90 complex formation is dependent on CD167a kinase, the selective CD167a/DDR1 inhibitor (DDR1-IN-1) was used and found to partially disrupt this complex formation (Fig. 6f, g). Since HSP90 was reported elsewhere to be a regulator for Stat3^39,40^; accordingly, we explored whether disruption of HSP90 function by its potent inhibitor, Geldanamycin, might affect the tyrosine phosphorylation of Stat3. Geldanamycin effectively disrupted the interaction of HSP90 with CD167a in the T24 model (Fig. 6f), and convincingly diminished ASMC-induced Stat3 tyrosine phosphorylation in a dose-dependent manner, both in T24 bladder cancer (Fig. 6h) and the human PDX models (Fig. 6i). Similarly, COL3-induced Stat3 tyrosine phosphorylation was also significantly abrogated by the HSP90 inhibitor (Fig. 6j, k). Collectively, these results uncovered CD167a-HSP90 as a newly-identified interacting protein complex, which connected Stat3 as a downstream molecule to the CD167a canonical axis in bladder cancer.

### Targeting Stat3 abrogates metastatic colonization in lung

**Fig. 7:**
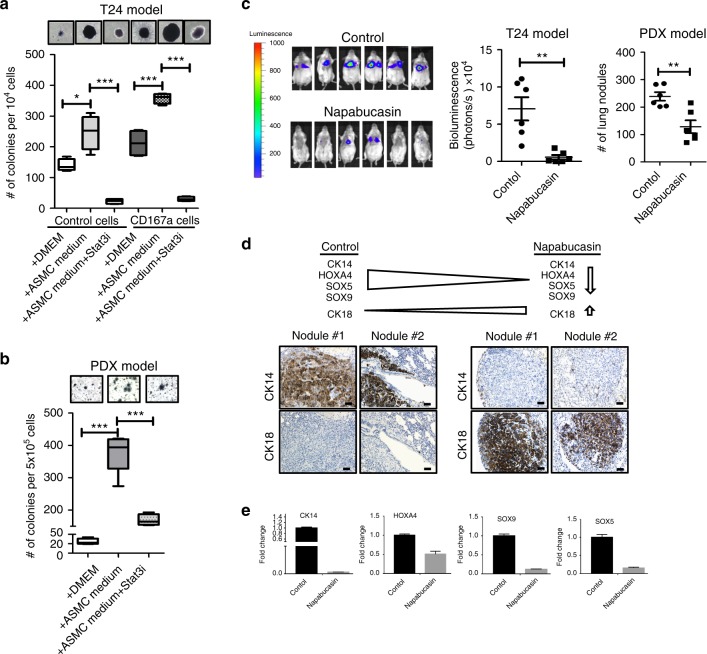
Stat3 as a target to abrogate lung colonization. **a**, **b** Colony forming efficiency of T24 and metastatic PDX model upon treatment with Napabucasin (Stat3 inhibitor, 1μM) in ASMC-conditioned media. Full colonies were allowed to form for 14 days, before the administration of vehicle or Napabucasin for an additional 7-day period. Live colonies were stained with the MTT assay for quantifications. **c** Assessment of metastatic lung colonization in T24 and metastatic PDX models upon treatment with Napabucasin for six weeks. 1 × 10^6^ cancer cells were injected via tail-vein. Napabucasin treatment (intraperitoneal injection, 20 mg kg^−1^) started 72 h post-injection (T24 model, *n* = 6; PDX model, *n* = 6). BLI signals were measured weekly. BLI images at the experimental endpoint (after 6 weeks treatment) with respective quantitative intensities are shown. **d** Immunohistochemical analysis of stem/progenitor and differentiation markers (CK14 and CK18) in vehicle and Napabucasin treated lung sections from mice injected with T24 cancer cells via tail-vein. **e** Gene expression analysis of stemness markers (CK14, SOX5, SOX9 and HOXA4) in lung sections of vehicle and Napabucasin-treated mice tail-vein injected with T24 cancer cells. Statistical analysis: a two-tailed, unpaired student’s t-test. Error bar: mean ± SEM. ^*^p < 0.05, ^**^p < 0.01

Since DDR1-IN-1 is a compound not proven for pharmacological application in vivo and HSP90 is a molecular chaperone with many known client proteins additional to Stat3, we decided to place emphasis onto targeting the CD167a-HSP90 downstream effector—Stat3, with drugs emergent in late phase clinical trials. We utilized Napabucasin, a small molecule inhibitor originally discovered with selective specificity toward Stat3^41^. Napabucasin (BBI-608) is currently approved by the FDA as an anti-cancer drug in use for Phase II/III clinical trials (https://www.cancer.gov/about-cancer/treatment/clinical-trials/intervention/napabucasin) and had been shown to prolong the survival of patients with colorectal cancer tumors expressing Stat3^42^. To evaluate whether drug targeting of Stat3 could abrogate ASMC-induced colony formation, Napabucasin was evaluated and significantly suppressed ASMC-induced colony formation capacity of cancer cells (Fig. 7a, b). Moreover, to evaluate the in vivo efficacy of Napabucasin in modulating lung colonization, we adopted the tail-vein injection model (as in Fig. 3h–i). Mice were treated with Napabucasin (*n* = 6) or vehicle as control (*n* = 6) at 72 h after injection of cancer cells (when cancer cells started lung colonization–Fig. 3h). Napabucasin effectively and significantly abolished metastatic lung colonization, when compared to vehicle treated control mice, in two independent metastatic models of bladder carcinomas (Fig. 7c). Furthermore, it is interesting to note that Stat3 is an important gene for maintaining stemness, and Napabucasin was independently shown to block stem cell activity of cancer cells through targeting Stat3 activity^41^. In our models, Napabucasin treatment similarly reduced the stemness property of lung colonizing cancer cells, illustrated by a significant decrease in the expression of stemness genes, e.g. CK14, SOX5, SOX9, and HOXA4, and an increased expression of the differentiated cell marker, CK18 (Fig. 7d, e). Collectively, these results revealed Stat3 as an important survival signal to maintain lung colonization.

## Discussion

**Fig. 8:**
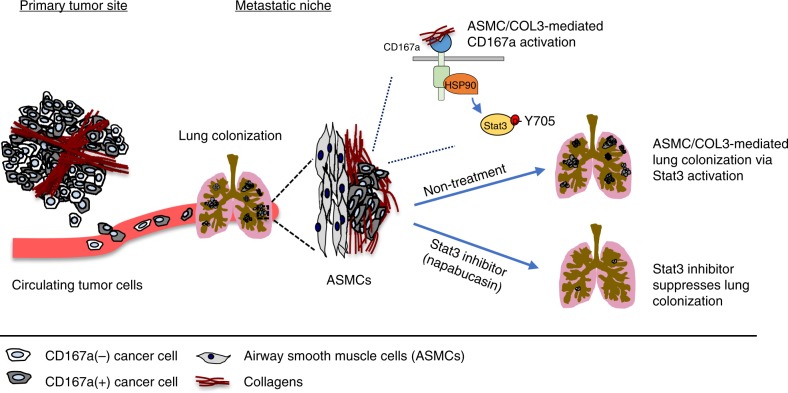
Schematic diagram illustrating different collagens in the primary tumor and metastatic microenvironment and their regulation of the metastatic cascade. Airway smooth muscle cells are a collagen III rich niche for metastatic colonization of bladder cancer cells expressing CD167a, and Stat3 is a downstream target for abrogating these collagen III/CD167a-driven metastatic foci

The tumor microenvironment is emerging as an important component that contributes to tumor progression and metastasis. While it is recognized that many cell types, including stromal cells, immune cells, and vasculatures constitute the tumor microenvironment^43–45^, our study here focused on investigating the roles of collagens and their crosstalk with cancer cells (Fig. 8). Collagens were previously thought to be passive scaffolds within the ECM that prevents cancer cells from early invasion into their surroundings. Emerging studies have revealed collagens as functional ligands that crosstalk with collagen receptors on neighboring cancer cells^46^; however, this crosstalk remains an unexplored territory for bladder carcinomas. Our current study reports collagen I expression within human primary bladder tumors positively correlates with increasing tumor staging–an independent prognostic marker and clinical parameter measuring the extent of invasion into muscle and perivesical tissues. Functionally, while exogenous collagen I consistently induced cell migration in monolayer cell cultures, its effect in a 3D culture was significantly more pronounced, provoking both unique sprouting and invasive morphologies of bladder cancer cells. Our findings are well supported by studies from other cancer types, demonstrating that collagen-CD167a signaling contributes to the invasive properties of cancer cells at the primary tumor site^47^.

While metastasis is a multi-step process, most studies linking collagen signaling to metastasis focuses on its roles during the initial steps of local invasion^15–17^. Data supporting a physical lung niche or microenvironment that facilitates metastatic colonization is not as well developed. While the thought-provoking premetastatic niche in lung was under extensive investigation^48,49^, to our knowledge, the pre-existence of a collagen niche that supports metastatic tumor colonization in the lung has not been explored. Our results guided us into this new territory, by unraveling a remarkable pathologic phenotype of CD167a-expressing bladder cancer cells and their metastatic colonization to airway smooth muscle cells (ASMCs) (Fig. 8), representing an original finding not reported elsewhere. Initial search of literatures within the cancer research field did not provide us with any clues foremost ASMCs as a potential niche for metastatic colonization. By expanding our search to other research topics, we uncovered that airway remodeling by ASMCs is pertinent to asthma models^29^. Increased ECM deposition by ASMCs is a mechanism associated with ASM hyperplasia and hypertrophy, contributing to various clinical symptoms of asthma, including airway obstruction, shortness of breath, and chest pain or tightness^30^. These symptoms are often associated in advanced stage metastatic patients with heavy lung metastatic burden (Canadian Cancer Society http://www.cancer.ca/), highlighting the clinical relevance of our results. Further, ASMCs are reported to deposit collagens as the major ECM components, and collagen III was reported as an abundantly expressed collagen type within lung tissues^31,32^. We therefore investigated collagen III deposition as a probable mechanism favoring the colonization of CD167a-expressing metastatic foci to ASMCs in lung; since collagen III is an alternative ligand for CD167a^33,34^. The role of collagen III in facilitating lung colonization was further reinforced by its capacity to activate the survival factor—Stat3. Pharmaceutical inhibition of Stat3 by Napabucasin—a drug already in Phase II/III clinical trials—significantly abrogated the capacity of CD167a-expressing cancer cells to form lung metastases. Others’ and our previous studies independently revealed Stat3 expression as a hallmark of bladder cancer patients with the basal molecular subtype^50,51^. These findings present the rationale to evaluate anti-Stat3 drugs in future clinical trials, selecting metastatic bladder cancer patients with basal phenotype as the target population. This is clinically significant, since bladder cancer is the second most common urologic malignancy affecting men (causes 11,510 death) after prostate cancer (27,540 death; 2015 data from American Cancer Society). And current clinical prospects for metastatic bladder cancer patients are devastating, with a 6–8% 5-year survival and limited treatment options^11–13^. While the newly FDA-approved immune checkpoint therapies created initial excitements for localized^52^ and invasive bladder cancers^43,53^, they recently missed the expected endpoints in a Phase III clinical trial. This is interestingly and timely, since a recent study elegantly posed DDR2 (a family member of CD167a/DDR1) as a valid target to complement with anti-PD-1 immunotherapy^54^. Moreover, our data here also highlights the importance of organ-site specific research, a recent report using breast cancer models indicated an alternative non-canonical mechanism^36^, our data in bladder carcinomas implicated the canonical CD167a/DDR1 pathway for activating Stat3. Using proteomic discovery approach, we discovered HSP90 as an interacting protein with CD167a in our bladder cancer model. Blockade of CD167a kinase activity not only disrupted its interaction with HSP90, but also reduced Stat3 phosphorylation^55–57^—revealing HSP90 as part of the canonical CD167a/DDR1 signaling. Since HSP90 is a molecular chaperone that regulates many client proteins, this poses another interesting future research direction.

Additionally, our results here provide important insights to explain why the collagen receptor downstream signaling might be a good therapeutic target for the management of distant metastases^36,58^. Metastatic bladder cancers are known to be unresponsive to cisplatin-based chemotherapy^11–13^, which could emerge from chemo-resistant clones within the primary tumors. Our recent report revealed collagen I deposition at the primary tumor as a hallmark of chemorefractory bladder cancer^59,60^, implicating a possible mechanism linking chemoresistance to metastatic progression. While it is somewhat intriguing that CD167a did not play a major role in other steps of the metastatic process, e.g. intravasation into circulation and extravasation from the circulation, our current study underscores the importance to carefully dissect each step of the metastatic cascade during mechanistic investigations. Together, our findings established a paradigm that CD167a-expressing cancer cells interacts with collagen I at the primary tumors to facilitate local invasion, and colonizes at collagen III-rich airway smooth muscle cells in lung (Fig. 8). While we recognize that other collagens are also expressed within these sites, our conception and recognition of the collagen microenvironments at the local and metastatic niche is timely. Recent exome sequencing of matched primary tumors and distal metastases from the same urothelial carcinoma patients remarkably revealed that they clonally evolved in parallel, resulting in tumor clones branching into distinct gene mutation patterns^61^. While no explanations were provided in that study, it seems plausible that distinct microenvironments at the local and metastatic sites likely contribute to such parallel progression of primary and metastatic tumor cells. Our data here provides insights to further investigate tumor-ECM interaction in the clonal evolution of primary and metastatic tumor cells in future studies. Our findings also revealed a generalized importance to convey ECMs and possibly other factors secreted by this ASMC metastatic niche to a broader audience for evaluation in other cancer types amid future studies.

## Methods

### Cell culture

The human bladder cancer cell line T24 was purchased from ATCC (HTB4). The highly metastatic T24 lung subline (LungMet line) was established from parental T24 cancer cells by serially re-injected (5 times) through tail-vein. Both cell lines were maintained in DMEM medium supplemented with 10% fetal bovine serum containing 100 μg ml^−1^ of streptomycin and 100 Units/ml of penicillin. Normal human primary airway smooth muscle cell (ASMC) line was purchased from ATCC (ATCC® PCS-130-010™) and cultured in vascular cell basal medium (ATCC® PCS-100-030™) supplemented with vascular smooth muscle cell growth kit (ATCC® PCS-100-042™). All cells were free of mycoplasma contamination by PCR examination and incubated at 37 °C in a humidified atmosphere containing 5% CO_2_. To monitor cancer cell growth, CTCs and lung metastasis in vivo, T24 cells were labeled with a dual-fusion reporter vector expressing mCherry and click-beetle luciferase (mCherry-CBLuc-T24 cells). For CD167a overexpression experiments, pHAGE-V5 or pHAGE-V5-CD167a plasmids were used. CD167a/DDR1 contains five isoforms (a, b, c, d, and e), b isoform of CD167a/DDR1 is the most abundant isoform and was used in the current study. These plasmids were packaged in 293T cells by co-transfection with compatible packaging plasmids. Culture supernatants containing the viral particles were collected 48 h post transfection and concentrated via high-speed centrifugation. mCherry-CBLuc-T24 cells were transduced with lentivirus-containing supernatant in the presence of 10 μg ml^−1^ polybrene for 24 h and replaced with selection medium containing 5 μg ml^−1^ puromycin for two weeks. The bladder PDX model was created by our laboratory from a pathologic T2 bladder cancer patient under the Institutional Animal Care and Use Committee (IACUC) protocol number H-25099. This human PDX tissue was ectopically embedded into immunocompromised mice, followed by an engraftment period of approximately two months, lung metastases were consistently observed. To culture the human bladder PDX line, DMEM/F12 medium supplemented with 10% fetal bovine serum containing 5 ml GlutaMAX™ solution, 20 ng/ml EGF, 10 mM HEPES, 1X Insulin-Transferrin-Selenium (ITS-G), Gentamicin and 1.5 g/L D-glucose was used. For the ASMC-conditioned medium experiments, vascular cell basal medium was removed from ASM cells, washed with PBS twice and subsequently cultured in DMEM medium overnight (16 h). The next day, ASMC-conditioned medium was transferred to T24 cancer cells or human bladder PDX line for another 24 h; cells lysates were then harvested for subsequent analysis.

### The clinical relevance of Collagen I gene expression in bladder cancer patients

The correlation between collagen I (*COL1A1* and *COL1A2*) gene expression and the invasive properties (or clinical staging) of human bladder cancer specimens was evaluated by using three independent cohorts of bladder cancer patient samples [kim, *n* = 165^20^, Riester, *n* = 93^21^, and TCGA, *n* = 376^22^].

### Antibodies and reagents

The following primary antibodies and dilutions were used: anti-CD167a at 1:1000 (WB) and 1:100 (IHC and IF) (Cell Signaling Technology, #5583), anti-Stat3 at 1:1000 (WB) and 1:100 (IHC) (Cell Signaling Technology, #9139), anti-phospho-Y705 Stat3 at 1:1000 (WB) (Cell Signaling Technology, #4113), anti-DNA-PKcs (G-4) at 1:1000 (Santa Cruz Biotechnology Inc., sc-5282); anti-GAPDH at 1:1000 (Santa Cruz Biotechnology Inc., sc-32233); anti-HSP90α/β (F-8) at 1:1000 (Santa Cruz Biotechnology Inc., sc-13119); anti-Collagen I at 1:100 (IHC) (Abcam ab34710), anti-Collagen III at 1:100 (IHC) (Abcam, ab7778), anti-mCherry at 1:100 (IHC) (Abcam ab167453) and anti-α-SMA at 1:400 (IF) (SIGMA, #A2547). Secondary antibodies were purchased from the following sources: for immunohistochemistry, anti-mouse and anti-rabbit horseradish peroxidase (HRP)–linked secondary antibodies were from Dako. For western blot analysis, anti-rabbit and anti-mouse HRP-conjugated secondary antibodies were from Cell signaling. For immunofluorescence analysis, goat anti-rabbit Alexa Fluor 488 (Z25302) and goat anti-mouse Alexa Fluor 594 (Z25207) were from Invitrogen. CD167a inhibitor, DDR1-IN1 was purchased from Selleckchem (S7498); Stat3 inhibitor, Napabucasin was purchased from Selleckchem (S7977); HSP90 inhibitor, Geldanamycin was purchased from Selleckchem (S2731). Protein agarose A/G beads for immunoprecipitation assays were purchased from Santa Cruz Biotechnology Inc., sc-2003. For collagen treatment, Collagen I was from purchased Corning (#354236) and Collagen III was from Advanced BioMatrix (#5021).

### Mass spectrometry-based immunoprecipitation (IP-MS) proteomics analysis

To identify DDR1/CD167a interacting proteins, cell lysates were collected from CD167a-overexpression T24 cells and using protein agarose A/G beads with anti-CD167a antibody to perform immunoprecipitation assay. After 4 h incubation at 4 °C, beads were washed with RIPA buffer for 4 times, and then IP samples were subjected to mass spectrometry analysis. Selected CD167a associated gene protein products (GPs) relative amounts from affinity purified samples was calculated using iBAQ algorithm by in-housed data processing algorithm. Simply, iBAQ was calculated based on normalization of summed peptide intensity (area-under-cureve) of precursor ion MS signal divided by the number of theoretically observable tryptic peptide of certain protein.

### Co-immunoprecipitation assay

Cell lysates were collected from T24 cells and human bladder PDX cultures treated with either COL3 (25 μg ml^−1^) or ASMC-conditioned media, in the presence or absence of DDR1-IN1 (10 μM) or Geldanamycin (5 μM) for 24 h. Five hundred micrograms of protein lysate (per sample) was incubated with 3 μg of anti-HSP90 antibody and 20 μl of protein agarose A/G beads at 4 °C for 4 h. Afterwards, beads were washed with RIPA buffer for four times, and then IP samples were subjected to western blot analysis.

### Western blot analysis

Cell pellets or xenograft tissues were lysed in RIPA buffer (EMD Millipore) with complete protease and phosphatase inhibitor cocktails (Thermo Scientific). Lysates were collected by centrifugation at 18407 × *g* for 15 min at 4 °C, and protein concentrations were measured by BCA assay. Twenty-five micrograms of sample lysates were subjected to western blot analysis using 4–12% Tris-Glycine gel under reducing conditions. Proteins were transferred onto PVDF membranes and probed with primary antibodies, anti-CD167a, Stat3, phospho-Y705 Stat3, HSP90α/β, and GAPDH were used at 1:1,000 dilution for standard immunoblotting with appropriate secondary HRP-conjugated antibodies (1:10,000 dilution). The bands were visualized using the enhanced chemiluminescence (ECL) system. Uncropped gel images are available in the Source Data.

### Mass spectrometer analysis of ASMC conditioned medium

Parallel reaction monitoring (PRM) was implemented to validate the amount of collagen III in ASMC conditioned medium. The conditioned medium from ASM cells were collected at 0 h, as control (incubated with ASM cells for 30 sec) and 16 h (after incubated with ASM cells for 16 h), and subsequently subjected to mass spectrometeric analyses. We utilized the PRM method using Orbitrap Fusion™ Tribrid™ mass spectrometer. Depends on unique peptide availability, three or four unique peptides for each target protein was selected for PRM analysis. Pre-selected precursor ions were scanned with a 10 min predicted elution window and isolated by quadrupole followed by collision-induced dissociation MS2 analysis. For relative quantification, the raw spectrum file was crunched to.mgf format by PD1.4 and then imported to Skyline with raw data file. We validated each result by deleting non-identified spectrum and adjusting the AUC range. Finally, the sum of the area of the three to four strongest product ions for each precursor ion (each peptide) were used for the result.

### Reverse transcription-PCR (RT-PCR) and quantitative real-time RT-PCR

Total RNA of airway smooth muscle cells and T24 cells were isolated and reverse-transcribed using the Aurum Total RNA mini kit (Cat. #732-6820, BIO-RAD) and Reverse Transcription Supermix kit (Cat. #1708841, BIO-RAD), respectively. The cDNA products were used to determine the mRNA expression of type III collagen with the primers list below. Quantitative real-time RT-PCR was performed by iTaq Universal SYBR Green Supermix (BI0-RAD, Cat. #172-5121) and Roche LightCycler96 machine. The abundance of mRNA of type III collagen was normalized to GAPDH abundance.

GAPDH: Forward:5′-TGCACCACCAACTGCTTAGC-3′

Reverse:5′- GGCATGGACTGTGGTCATGAG-3′

Collagen III: Forward: 5′-TCTTTGAATCCTAGCCCATCTG-3′

Reverse: 5′-TGTGACAAAAGCAGCCCCATAA-3′

For the examination of collagen I receptors mRNA expression upon collagen I treatment, T24 cells were treated with or without 25 μg  ml^−1^ collagen I for 24 h. Control and collagen I-treated cells were harvested and subjected to Quantitative real-time PCR using the following primers:

CD167a: Forward:5′- CCTGGGGACACTATCCTCATC-3′

Reverse:5′- GGATTGGAGAGCAGCAACG-3′

DDR2: Forward:5′- GCTATATGCCGCTATCCTCTGG-3′

Reverse:5′- ACTCTGACCACTGACTGGAAG-3′

ITGA1: Forward:5′- ACGCTGCTGCGTATCATTCA-3′

Reverse:5′- CAAACATGTCTTCCACCGGG-3′

ITGB1: Forward:5’- GTAACCAACCGTAGCAAAGGA-3’

Reverse:5′- TCCCCTGATCTTAATCGCAAAAC-3′

ITGA11: Forward:5′- GTCACCCTGTCCAACGTGTC-3′

Reverse:5′- ACATCCCTGTGGTGTAGTAGG-3′

MRC2: Forward:5′- ACATCACCATGGGTGTCGTC-3′

Reverse:5′- GGTTCTCTGGAAGCGCTGAT-3′

To examine the stemness-associated genes expression in control and Napabucasin-treated lung nodules, formalin-fixed, paraffin-embedded (FFPE) lung sections were cut, collected and used High Pure FFPET RNA Isolation Kit (Roche, REF06650775001) to extract RNA and using the following primers for Quantitative real-time PCR:

CK14: Forward:5′- CTCACAGCCACAGTGGACAA-3′

Reverse:5′- TCATGCGCAGGTTCAACTCT-3′

CK18: Forward:5′- TATCACACGACTGCAGCTGG-3′

Reverse:5′- TGGCAATCTGGGCTTGTAGG-3′

HOXA4: Forward:5′- ATAACGGAGGGGAGCCTAAG-3′

Reverse:5′- GCTCAGACAAACAGAGCGTG-3′

SOX5: Forward:5′- CAGCCAGAGTTAGCACAATAGG-3′

Reverse:5′- CTGTTGTTCCCGTCGGAGTT-3′

SOX9: Forward:5′- AGCGAACGCACATCAAGAC-3′

Reverse:5′- CTGTAGGCGATCTGTTGGGG-3′

### Two-dimensional wound-healing/scratch assay

For in vitro migration studies, cells were plated in triplicate on 96-well Essen Image Lock plates (Essen BioScience) at 40,000 cells per well. Cancer cells were pre-treated with 30 ng/ml Mitomycin C for 2 h to block cell proliferation. The Essen Wound Maker (Essen BioScience) was used to make the wounds in confluent cell culture monolayers. Cancer cells were then treated with either 25 or 50 μg ml^−1^of collagen I (Invitrogen) after scratch. Wound closure was monitored by acquiring images every 1 h over a 24 h period with the Incucyte ZOOM. An integrated metric called relative wound density (RWD) was used to quantify effects on migration. The grey area indicates the “wound gap” (devoid of cells), while the orange area indicates the movement of cancer cells migrating toward the gap area.

### Three-dimensional cell culture and invasion assay

To determine whether collagen I can alter cells growth behavior in a 3D cell culture condition, a total of 2000 T24 cancer cells were cultured in Matrigel matrix (growth-factor reduced) mixed with or without collagen I (0.25 mg ml^−1^) and subsequently plated onto 96-well plates. Cancer cells were allowed to grow into colonies for an additional 14 days. Concurrently, new culture medium was added every 2 days to avoid the matrigel matrix drying. To mimic the local cell invasion in vivo, 3D collagen type I invasion assays were performed, the top gel was embedded with cancer cells at high density, in the absence or presence of increasing collagen I concentrations, while the bottom gel constituted an empty matrix to allow cancer cells to invade through. The collagen gels mixed with Matrigel (*n* = 3 gels per condition, 60 μl volume) were prepared at a final concentration of 2.5 mg ml^−1^, and placed in a 96-well plate. The collagen gels were allowed to polymerize and equilibrate at 37 °C and 5% CO_2_ for 30 min. After equilibration, 60,000 cells per well were seeded onto the surface of the gel and subsequently treated with the indicated concentration of collagen I (0, 10, and 25 μg ml^−1^), and then allowed to invade 3D collagen gels for 72 h. At the time of collection, culture medium was removed and collagen matrices containing invading cancer cells were fixed in 4% PFA for 20 min. Matrices were stained with 0.1% toluidine blue in 30% methanol for 30 min prior to washing with water.

### Immunohistochemistry (IHC) and immunofluorescence (IF) analysis

Tumor and lung tissues were collected from mice, and embedded in paraffin after formalin fixation at room temperature. For IHC analysis, tissue sections were heated at 65 °C for 60–90 min, deparaffinized in xylene, rehydrated in an ethanol gradient, submerged in citrate buffer (pH 6.0) and heated for 25 min to unmask antigens. Following a 3% H_2_O_2_ treatment for 10 min to block endogenous peroxidase activity, serial sections were incubated with primary antibodies for 90 min at room temperature, washed with TBST buffer three times and then incubated with horseradish peroxidase (HRP)-conjugated secondary antibodies for 60 min at room temperature. Nuclei were counterstained with hematoxylin. For the IF analysis, the staining protocol was similar to IHC, after 90 min primary antibodies incubation, the tissue slides were washed with TBST buffer three times and then incubated with fluorescence-conjugated secondary antibodies (goat anti-rabbit Alexa Fluor 488 and goat anti-mouse Alexa Fluor 594) for 60 min at room temperature. Nuclei were counterstained with DAPI.

### In vitro soft agar colony formation assay

The bottom of a six-well plate was coated with 1.5 mL of 0.7% low melting agarose, which was then covered with 1.5 mL of 0.35% agarose containing either 10,000 vector-control or CD167a-overexpressing mCherry-CBLuc T24 cells, or 5 × 10^5^ of the human bladder PDX cells. To determine the effect of Collagen III on colony formation, 0.35% agarose gel was mixed with 50 μg ml^−1^ Collagen III. Regularly, the six-well plates were incubated for 14 days at 37 °C with 5% CO_2_. DMEM medium was added every other day to the upper agar layer to prevent desiccation. To examine the effect of ASM cells on the colony formation of cancer cells, DMEM was replaced with ASMC-conditioned medium to add to the cells. For Stat3 inhibition experiments, 1 μM Napabucasin or vehicle was added to the cells at day 14 of the assay and incubated for an additional 7 days. Live colonies were stained with the MTT reagent (3-(4,5-dimethylthiazol-2-yl)-2,5-diphenyltetrazolium bromide) for 2 h and quantified at the end point.

### Xenograft tumor growth

A total of 1 × 10^6^ vector-control and CD167a overexpression mCherry-CBLuc T24 cells were injected into the 6 to 8 week-old Rag2^−/−^; IL-2Rγc^−/−^ immunocompromised mice. All animals were treated in accordance with IUCAC guidelines. The body weight of each mice and their corresponding tumor size were measured to observe how tumor progression impacted the health status of each mouse. Tumor diameters were measured at regular intervals with a caliper, and the tumor volume in mm^3^ was calculated weekly by the formula: volume = length × width × height. The tumors were isolated and snap-frozen in liquid nitrogen for western blot analysis or fixed with 3.7% formalin for immunohistochemical analysis at the end of experimental time point.

### Flow cytometry analysis

To examine the frequency of CTC, equal volume (100 µL) of whole peripheral blood was collected simultaneously from vector-control and CD167a-overexpressing mCherry-CBLuc mice. Red blood cells were lysed by the addition Ammonium Chloride solution (ACK), washed and then subjected to flow cytometry for single and clustered CTCs analysis. CTCs detection was based on fluorescent protein (i.e., mCherry) expression. Distinction between single and clustered CTCs was based on the side scattered (SSC)-area vs SSC-width plot.

### Tail-vein injection model to evaluate extravasation and lung colonization

To select for a highly lung metastatic T24 subline, 1 × 10^6^ mCherry-CBLuc-T24 cells were injected into mice by tail-vein. Tumor growth was monitored by bioluminescent signal using in vivo IVIS imaging. Mice carrying heavy lung metastatic burden were sacrificed and lung tissues containing metastatic foci were enzymatically dissociated for re-injected into mice via the tail-vein. This experimental selection was repeated five consecutive times.

To evaluate the role of CD167a during extravasation and/or colonization, 1 × 10^6^ vector-control or CD167a overexpression mCherry-CBLuc T24 cancer cells were injected into mice via tail-vein and extravasation was assessed measuring the bioluminescence signal intensity at 72 h post injection. Lung colonization was evaluated by monitoring the bioluminescence signal in the lung weekly for a total of 5 weeks. To examine the in vivo effect of the Stat3 inhibitor, Napabucasin, in modulating metastatic lung colonization, mice were treated with Napabucasin (20 mg kg^−1^, intraperitoneal injection) or vehicle control at 72 h post tail-vein injection of cancer cells. Mice were treated daily for 3 weeks with Napabucasin and then every other day for another 3 weeks, for a total of 6-weeks treatment.

### Statistics

Student’s two sample *t*-test was used to determine if there was statistical difference between the means of two groups (e.g. control and experimental groups). Analysis of Variance (ANOVA) was used for comparing three or more group means. *P*-values were two-sided and *p*-value < 0.05 was considered statistically significant. Quantified data shown represent at least three independent experiments. Data were represented as mean ± SEM.

### Study approval

Animal experiments were conducted according to a protocol reviewed and approved by the IACUC of Baylor College of Medicine.

### Reporting Summary

Further information on research design is available in the Nature Research Reporting Summary linked to this article.

## Supplementary information

Supplementary Information

Reporting Summary

Source Data

## Data Availability

The data that support the findings of this study are available from the corresponding author upon reasonable request.
