# Supplementary material for: Collagen-rich airway smooth muscle cells are a metastatic niche for tumor colonization in the lung
Source: Nat Commun. 2019 May 13;10:2131. doi: 10.1038/s41467-019-09878-4 (PMC6513865; doi:10.1038/s41467-019-09878-4)
Supplement: Supplementary file 1 — Supplementary Information [file 41467_2019_9878_MOESM1_ESM.pdf]

Supplementary Information

Collagen-rich airway smooth muscle cells is a metastatic niche for tumor colonization in the lung

Lee et al.

Contents

Supplementary Fig.1 to Fig.7

Supplementary Fig. 1

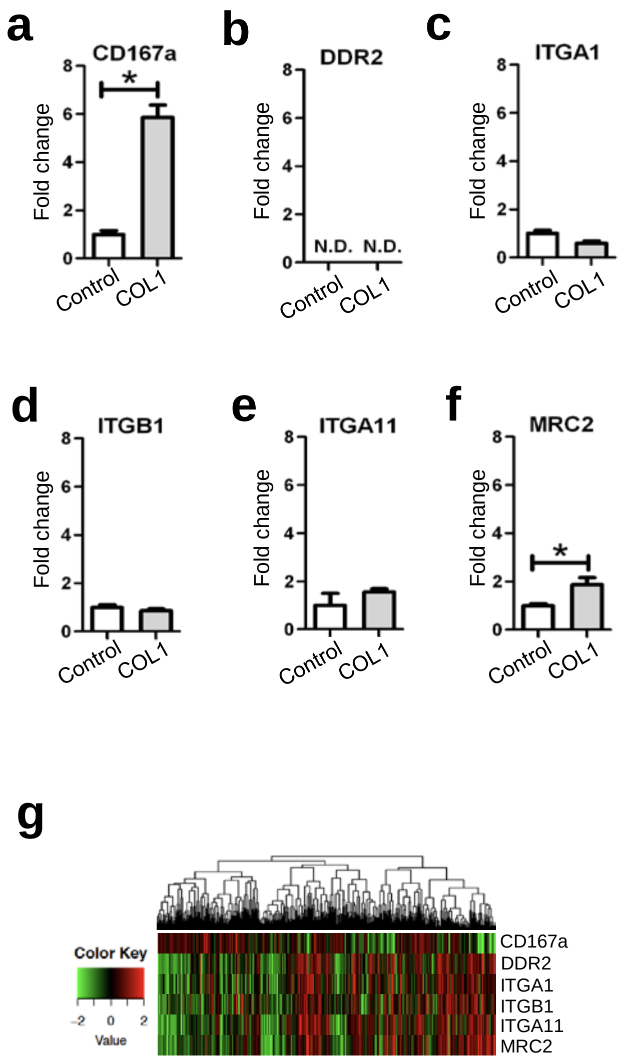

Supplementary Fig. 1 Collagen I treatment up-regulates CD167a mRNA expression. Bar graphs quantifying the relative mRNA expression of collagen I receptors in T24 cancer cells, in the presence or absence of collagen I treatment (25 μg ml^{-1}). **a** CD167a, **b** DDR2, **c** ITGA1, **d** ITGB1, **e** ITGA11 and **f** MRC2, which were normalized against GAPDH gene expression and control cells. ND: Non-detected. **g** A heat map depicting the expression of collagen receptor genes (CD167a, DDR2, ITGA1, ITGB1, ITGA11 and MRC2) in a human bladder cancer patient cohort; red and green colors indicate high and low expression, respectively. Statistical analysis: a two-tailed, unpaired student's t-test. Error bar: mean ± SEM. $^{*}p<0.05$

Supplementary Fig. 2

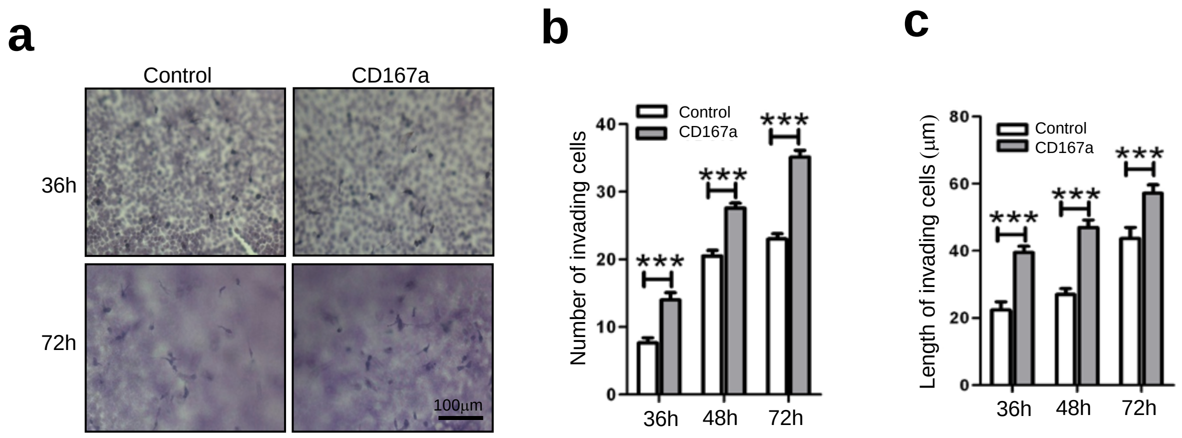

Supplementary Fig. 2 CD167a overexpression enhances cancer cell invasive capacity in 3D matrigel. Vector-control and CD167a-overexpressing mCherry-CBLuc-T24 cancer cells were subjected to the same 3D gel invasion assay as described in **Fig. 1f** and **1g**. Representative photos of indicated groups at the relative time points were captured in **a**. The number and distance of invading cells from the monolayer into the matrix were quantified as graphs in **b** and **c**, respectively. Statistical analysis: a two-tailed, unpaired student's t-test. n=3 independent experiments. Error bar: mean ± SEM. $^{***}p$<0.001

Supplementary Fig. 3

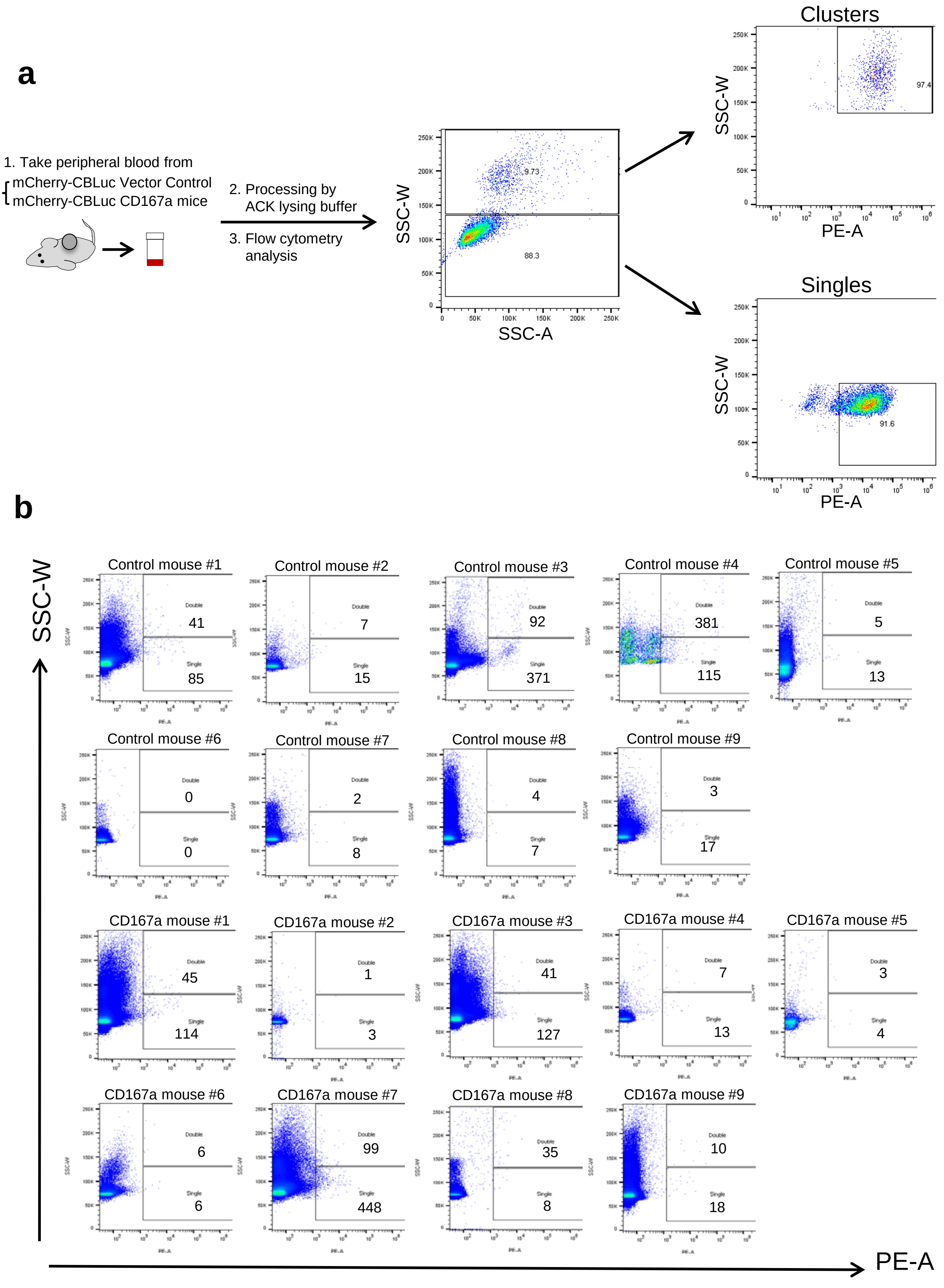

Supplementary Fig. 3 Quantification of single and clustered Circulating Tumor Cells. **a** The gating strategy for single and clustered CTCs were drawn, based on the side scattered (SSC)-area vs SSC-width plot as indicated. CTCs detection was based on fluorescent protein (i.e., mCherry) expression on the PE channel. **b** Whole peripheral blood (100μl) were collected from immunocompromised mice carrying xenograft tumors derived from control or CD167a-overexpressing mCherry-CBLuc-T24 cancer cells inoculation. Peripheral blood samples were first deprived of red blood cells, and then analyzed for single and clustered CTCs using flow cytometry.

Supplementary Fig. 4

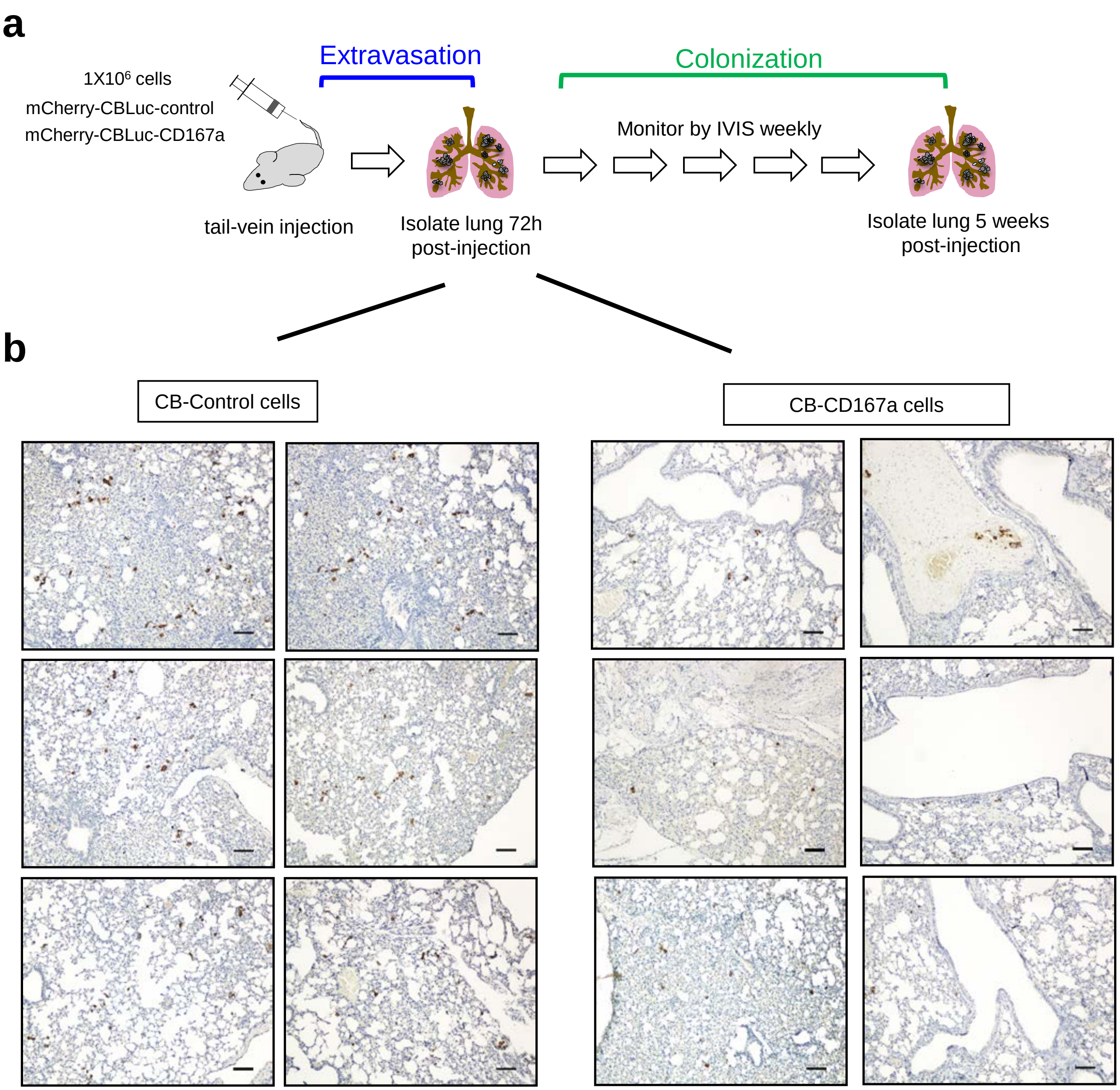

Supplementary Fig. 4 CD167a does not enhance extravasation from the circulation into lung. **a** A schematic diagram illustrating the tail-vein injection model is present for the evaluating extravasation of cancer cells from the circulation, since blood vessels in lung are the first capillary bed to be encountered. $1x10^6$ mCherry-CBLuc vector control (CB-Control) or CD167a-overexpressing (CB-CD167a) T24 cancer cells were injected into the tail-vein. At 72 hours after injection, serial sections of lung tissues were evaluated via IHC to quantify the frequency of extravasated cancer cells. **b** Representative lung tissues showing extravasated cancer cells from CB-control and CB-CD167a. Lung tissue sections were evaluated via IHC analysis by anti-mCherry antibody to outline cancer cells. CD167a-overexpressing T24 cells exhibited a unique localization near or within ASMCs at such early stage.

Supplementary Fig. 5

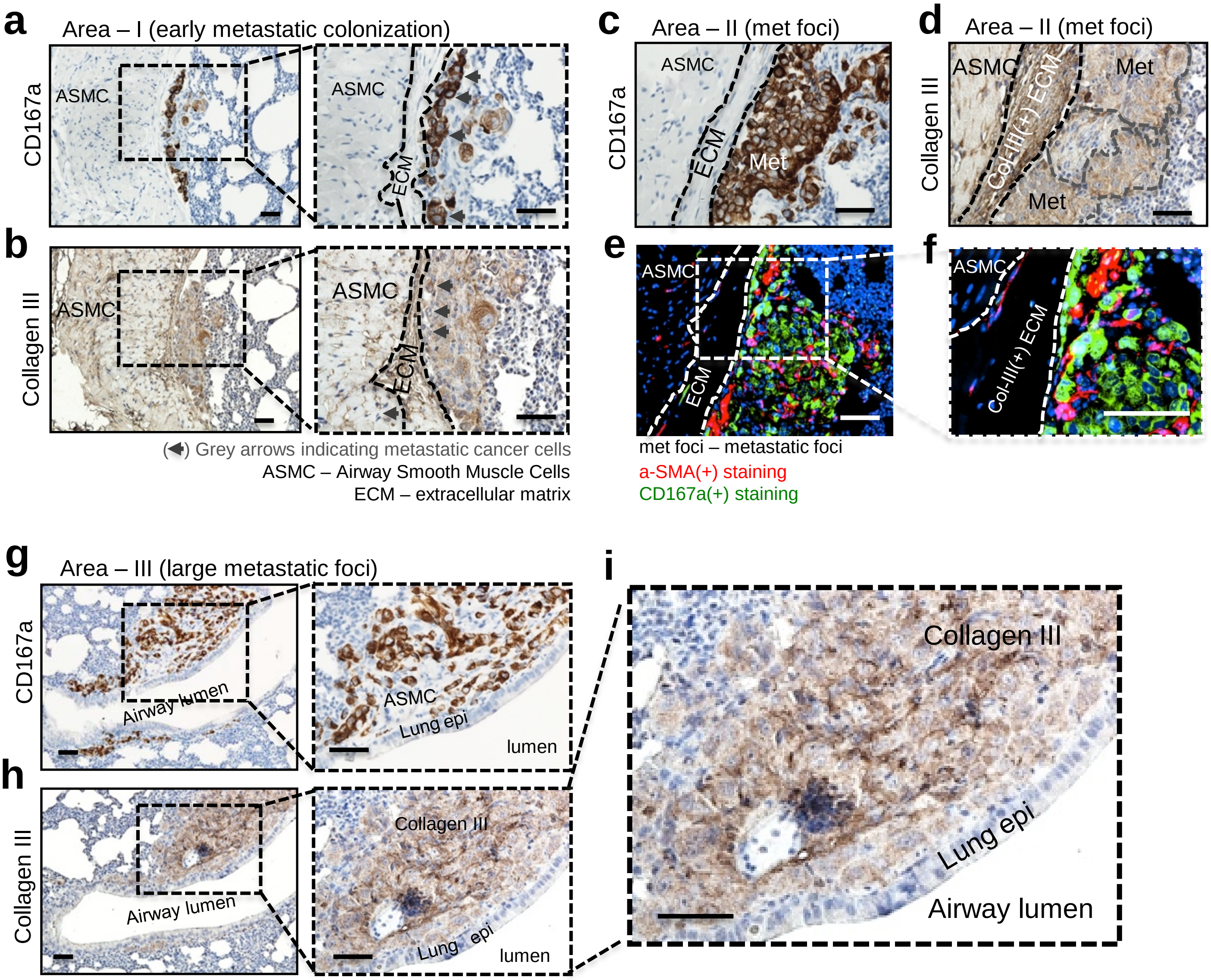

Supplementary Fig. 5 Collagen-rich airway smooth muscle cells as a metastatic niche. Photos demonstrating the colonization of CD167a-overexpressing cancer cells within COL3-rich airway smooth muscle cells (ASMC). Immunohistochemical staining using anti-CD167a and anti-collagen III antibodies in serial lung sections to analyze metastatic foci formed from CD167a-overexpressing cancer cells. **a** to **f** Area I and II indicate sites of early colonization, i.e. small cluster of cancer cells aligning in parallel along COL3-rich ECM. **g** to **i** Area III indicates larger metastatic nodule encapsulated by COL3-rich ECM. Negative areas of alveoli indicate specificity of COL3 staining. (ASM-Airway smooth muscle; ECM-extracellular matrix; epi-epithelium).

Supplementary Fig. 6

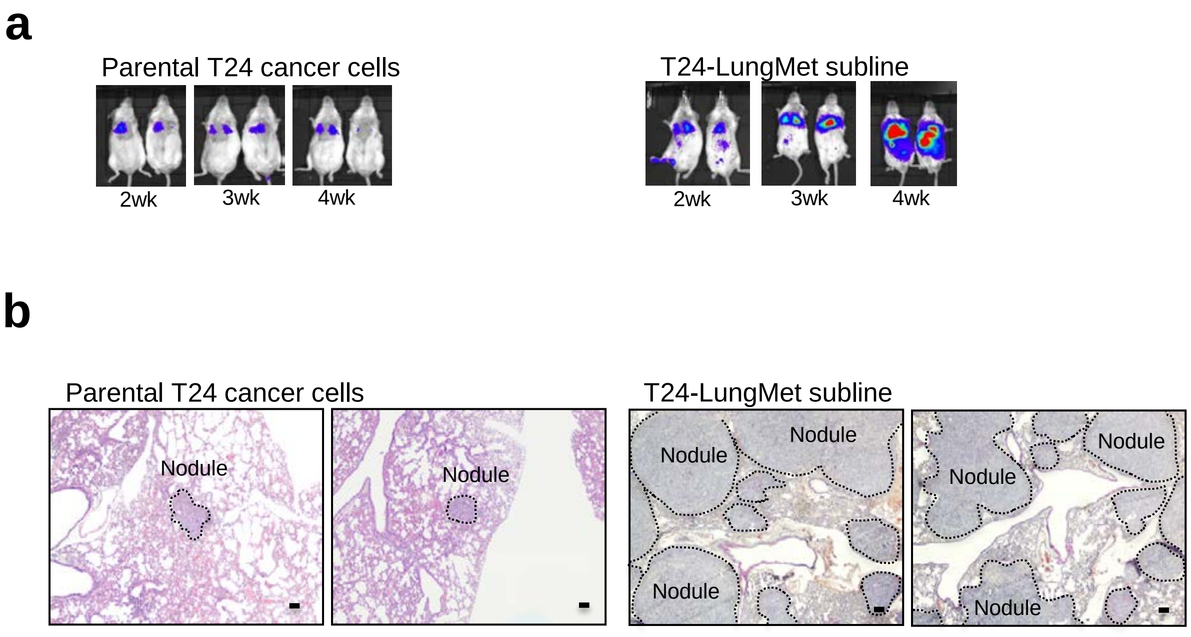

Supplementary Fig. 6 Establishment of a T24-LungMET subline with enhanced capacity to colonize lung. **a** Bioluminescence analysis indicating shorter latency of lung colonization by T24-LungMET subline. Either $1x10^6$ luciferase conjugated-parental T24 cancer cells or T24-LungMET subline were injected into mice by tail-vein injection, and subsequently tracked for lung colonization using IVIS signaling (weekly) for 4 weeks. **b** Lung sections were collected from parental T24 cancer cells and T24-LungMET subline 4 weeks post injection, and then analyzed by H&E staining. Representative images of H&E staining illustrated significant enhancement of metastatic lung colonization by the T24-LungMET subline; both in number and size. Scale bar:100mm.

Supplementary Fig. 7

GPPGLAGAPGLR

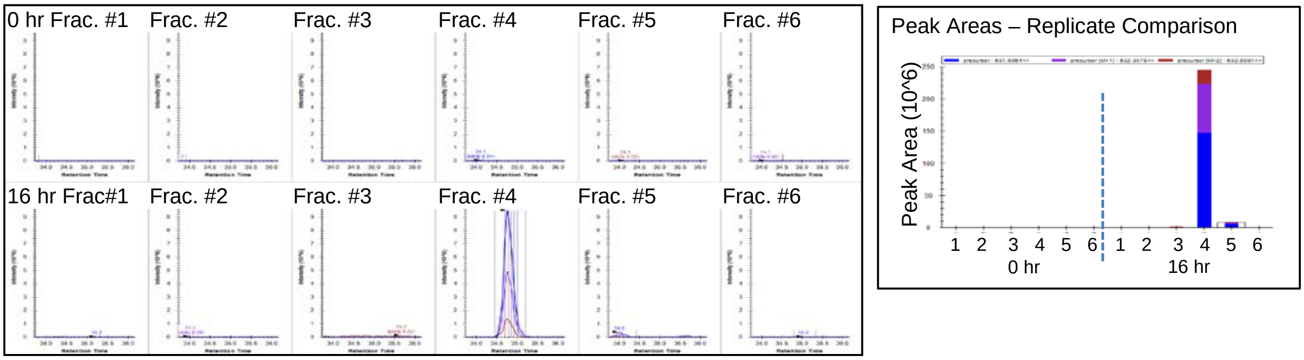

AGGFAPYYGDEPMDFK

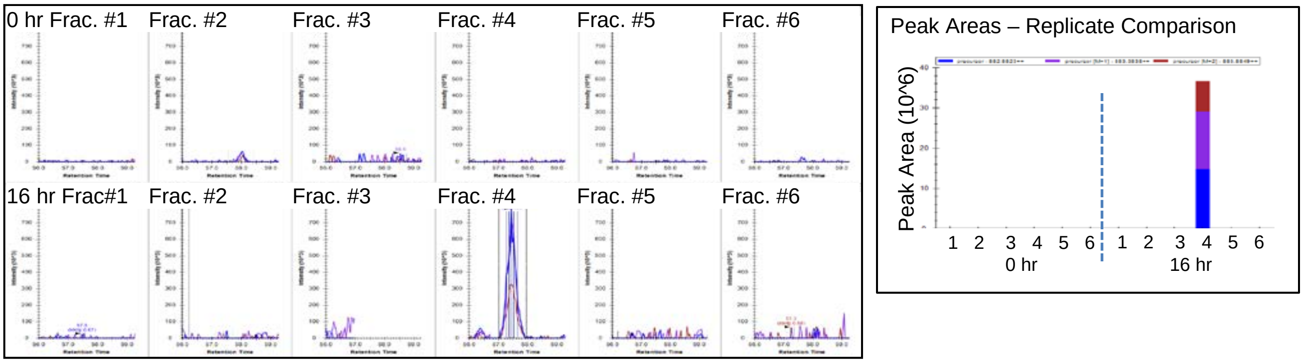

INTDEIMTSLK

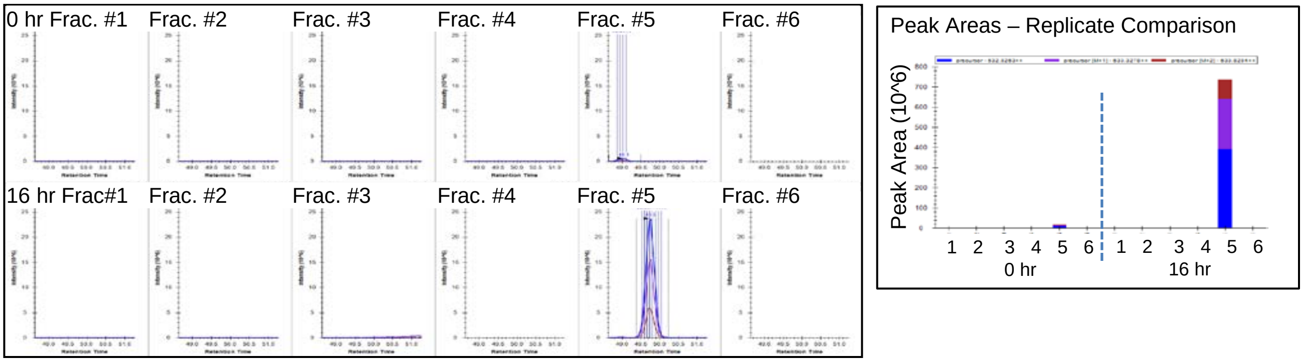

DGNPGSDGLPGR

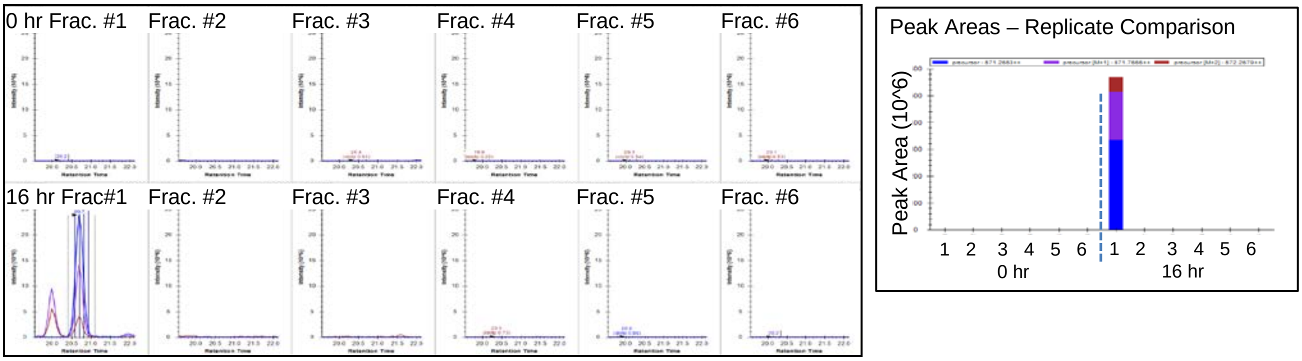

Supplementary Fig. 7 COL3A1 is abundantly secreted by cultured ASM cells in the conditioned medium. Parallel reaction monitoring for four selected COL3A1 peptides, GPPGLAGAPGLR, AGGFAPYYGDEPMDFK, INTDEIMTSLK and DGNPGSDGLPGR was carried out using Orbitrap Fusion™ Tribrid™ mass spectrometer (Thermo Scientifc™). Precursor ions (full mass) were scanned with 120,000 of resolution and $2.0e^5$ of AGC target and 50mSec maximum injection time using Orbitrap. Target precursor ions were isolated by Quadrupole with an isolation width of 2 m/z with 6 min predicted elution window. Product ions (MS2) were generated by CID at 35% Collison energy and scanned at 350-1400 m/z with $5.0e^4$ of AGC target and maximum 35mSec injection time in Orbitrap with 30,000 resolutions. The raw spectrum file was crunched to .mgf format by PD1.4 and then imported to Skyline together with raw data file. The amount of COL3A1 was calculated based on MS1 signal area-under curve using SKYLINE software.
