# Supplementary material for: Collagen-rich airway smooth muscle cells are a metastatic niche for tumor colonization in the lung
Source: Nat Commun. 2019 May 13;10:2131. doi: 10.1038/s41467-019-09878-4 (PMC6513865; doi:10.1038/s41467-019-09878-4)
Supplement: Supplementary file 2 — Reporting Summary [file 41467_2019_9878_MOESM2_ESM.pdf]

nature research

Corresponding author(s): Chan, keith Syson

Last updated by author(s): Mar 9, 2019

Reporting Summary

Nature Research wishes to improve the reproducibility of the work that we publish. This form provides structure for consistency and transparency in reporting. For further information on Nature Research policies, see Authors & Referees and the Editorial Policy Checklist.

Statistics

For all statistical analyses, confirm that the following items are present in the figure legend, table legend, main text, or Methods section.

n/a	Confirmed	
☐	☒	The exact sample size (*n*) for each experimental group/condition, given as a discrete number and unit of measurement
☐	☒	A statement on whether measurements were taken from distinct samples or whether the same sample was measured repeatedly
☐	☒	The statistical test(s) used AND whether they are one- or two-sided<br>*Only common tests should be described solely by name; describe more complex techniques in the Methods section.*
☐	☒	A description of all covariates tested
☐	☒	A description of any assumptions or corrections, such as tests of normality and adjustment for multiple comparisons
☐	☒	A full description of the statistical parameters including central tendency (e.g. means) or other basic estimates (e.g. regression coefficient) AND variation (e.g. standard deviation) or associated estimates of uncertainty (e.g. confidence intervals)
☐	☒	For null hypothesis testing, the test statistic (e.g. *F*, *t*, *r*) with confidence intervals, effect sizes, degrees of freedom and *P* value noted<br>*Give P values as exact values whenever suitable.*
☒	☐	For Bayesian analysis, information on the choice of priors and Markov chain Monte Carlo settings
☒	☐	For hierarchical and complex designs, identification of the appropriate level for tests and full reporting of outcomes
☒	☐	Estimates of effect sizes (e.g. Cohen's *d*, Pearson's *r*), indicating how they were calculated

Our web collection on statistics for biologists contains articles on many of the points above.

Software and code

Policy information about availability of computer code

Data collection	n.a.
Data analysis	n.a.

For manuscripts utilizing custom algorithms or software that are central to the research but not yet described in published literature, software must be made available to editors/reviewers. We strongly encourage code deposition in a community repository (e.g. GitHub). See the Nature Research guidelines for submitting code & software for further information.

Data

Policy information about availability of data

All manuscripts must include a data availability statement. This statement should provide the following information, where applicable:

- Accession codes, unique identifiers, or web links for publicly available datasets
- A list of figures that have associated raw data
- A description of any restrictions on data availability

The data that support the findings of this study are available from the corresponding author upon reasonable request.

Field-specific reporting

Please select the one below that is the best fit for your research. If you are not sure, read the appropriate sections before making your selection.

☒ Life sciences ☐ Behavioural & social sciences ☐ Ecological, evolutionary & environmental sciences

For a reference copy of the document with all sections, see nature.com/documents/nr-reporting-summary-flat.pdf

Life sciences study design

All studies must disclose on these points even when the disclosure is negative.

Sample size	For each experiment, we initially performed a smaller sample e.g. n=5 to determine whether experimental groups showed statistically significance, followed by consultation with bioinformatic collaborator to calculate the necessary sample size for future experiment for rigor and reproducibility. Therefore, throughout our manuscript, we have used at least n=5 to n=9 per experimental group for experimentation, and were sufficient to demonstrate statistical significance for the corresponding experiments performed.
Data exclusions	n.a
Replication	For each experiment, we have at least performed three repeats to ensure reproducibility
Randomization	We used age and sex matched animals, and randomized them into evenly distributed groups (if possible) for experimentation.
Blinding	When analyzing experimental results, data were collected or animals sacrificed by one individual (e.g. Jing Xiao) to maintain records and origin of data points. And subsequent evaluation and data analyzed were performed by a second experimenter (e.g. Yu Cheng Lee) blinded. Final data compilation were performed by connecting the blindly analyzed data with the original data collected.

Reporting for specific materials, systems and methods

We require information from authors about some types of materials, experimental systems and methods used in many studies. Here, indicate whether each material, system or method listed is relevant to your study. If you are not sure if a list item applies to your research, read the appropriate section before selecting a response.

Materials & experimental systems

n/a	Involved in the study	
☐	☒	Antibodies
☐	☒	Eukaryotic cell lines
☒	☐	Palaeontology
☐	☒	Animals and other organisms
☒	☐	Human research participants
☒	☐	Clinical data

Methods

n/a	Involved in the study	
☒	☐	ChIP-seq
☐	☒	Flow cytometry
☒	☐	MRI-based neuroimaging

Antibodies

Antibodies used	The following primary antibodies and dilutions were used: anti-CD167a at 1:1000 (WB) and 1:100 (IHC and IF) (Cell Signaling Technology, #5583), anti-Stat3 at 1:1000 (WB) and 1:100 (IHC) (Cell Signaling Technology, #9139), anti-phospho-Y705 Stat3 at 1:1000 (WB) (Cell Signaling Technology, #4113), anti-DNA-PKcs (G-4) at 1:1000 (Santa Cruz Biotechnology Inc., sc-5282); anti-GAPDH at 1:1000 (Santa Cruz Biotechnology Inc., sc-32233); anti-HSP90α/β (F-8) at 1:1000 (Santa Cruz Biotechnology Inc., sc-13119); anti-Collagen I at 1:100 (IHC) (Abcam ab34710), anti-Collagen III at 1:100 (IHC) (Abcam, ab7778), anti-mCherry at 1:100 (IHC) (Abcam ab167453) and anti-a-SMA at 1:400 (IF) (SIGMA, #A2547).
Validation	When possible, e.g. CD167a/DDR1 antibody, we overexpress and knockdown CD167 in a cell line to verify the specific reactivity of the antibody toward the target.

Eukaryotic cell lines

Policy information about cell lines

Cell line source(s)	T24 (ATCC -HTB4); airway smooth muscle cell from ATCC (ATCC® PCS-130-010™)
Authentication	We have sent passaged cell line back to ATCC for performing STR profiling to ensure its originality and authenticity
Mycoplasma contamination	no
Commonly misidentified lines (See ICLAC register)	n.a. cell lines ensured by STR profiling

Animals and other organisms

Policy information about studies involving animals; ARRIVE guidelines recommended for reporting animal research

Laboratory animals	Rag/Gamma chain double knock out mice

Wild animals	n.a.
Field-collected samples	n.a.
Ethics oversight	AN-5803, H-25099

Note that full information on the approval of the study protocol must also be provided in the manuscript.

Flow Cytometry

Plots

Confirm that:

☒ The axis labels state the marker and fluorochrome used (e.g. CD4-FITC).

☒ The axis scales are clearly visible. Include numbers along axes only for bottom left plot of group (a 'group' is an analysis of identical markers).

☒ All plots are contour plots with outliers or pseudocolor plots.

☒ A numerical value for number of cells or percentage (with statistics) is provided.

Methodology

Sample preparation	Peripheral blood were collected and RBCs were lysed by ACK, washed and subjected to flow cytometry (BD Fortessa) to analyze for fluorescent protein activity
Instrument	BD Fortessa
Software	BD FACSDIVA™ software for data collection, and FlowJO software for data analysis
Cell population abundance	CTCs were extremely rare as expected, representing only 0 to 448 CTCs depending on the individual sample per 100uL peripheral blood analyzed
Gating strategy	We used fluorescent protein transduced cells as positive gate, and empty-vector transduced cells (no fluorescence) as negative gate e.g. Supplementary Fig. 3 to determine the frequency of CTCs. SSC-W was used to determine cluster CTCs versus single CTCs

☒ Tick this box to confirm that a figure exemplifying the gating strategy is provided in the Supplementary Information.
