# Supplementary material for: Collagen-rich airway smooth muscle cells are a metastatic niche for tumor colonization in the lung
Source: Nat Commun. 2019 May 13;10:2131. doi: 10.1038/s41467-019-09878-4 (PMC6513865; doi:10.1038/s41467-019-09878-4)

Source data file to

Collagen-rich airway smooth muscle cells is a metastatic niche for tumor colonization in the lung

Lee et al.

Contents

Uncropped and unprocessed scans of Western Blots presented in Fig.2 to Fig.6

Fig. 2c

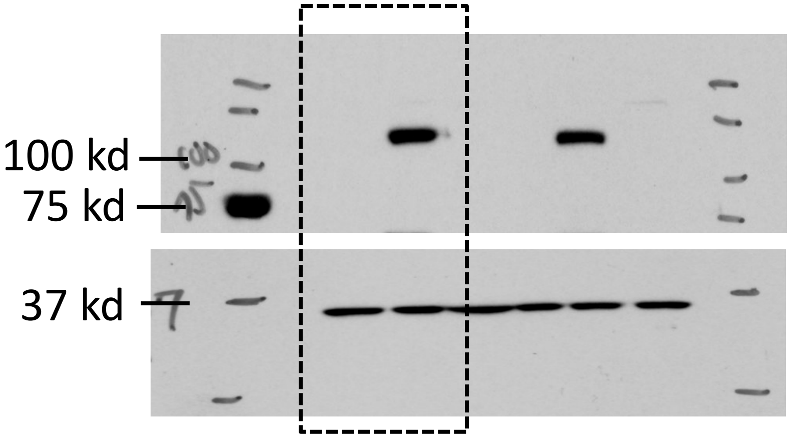

Fig. 2d

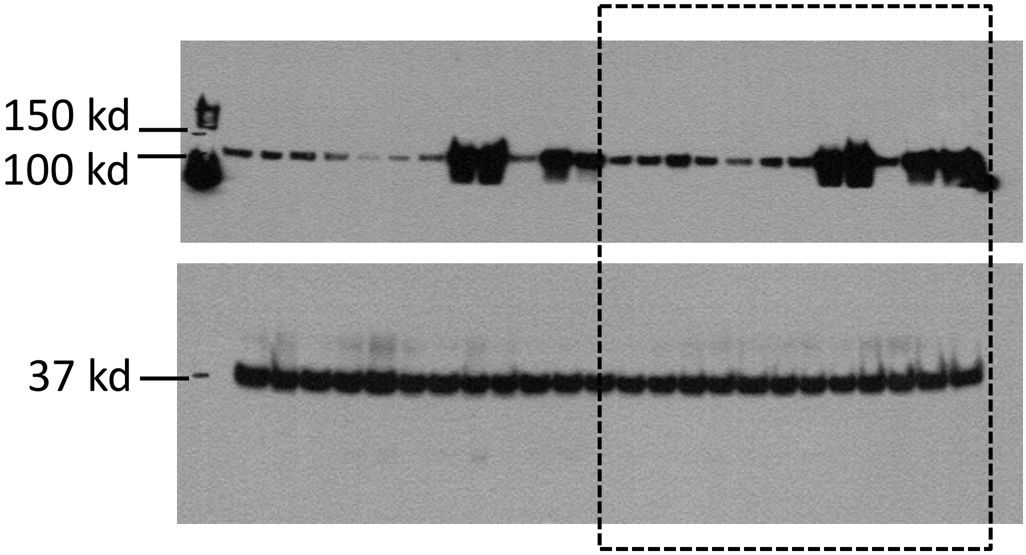

Fig. 4r

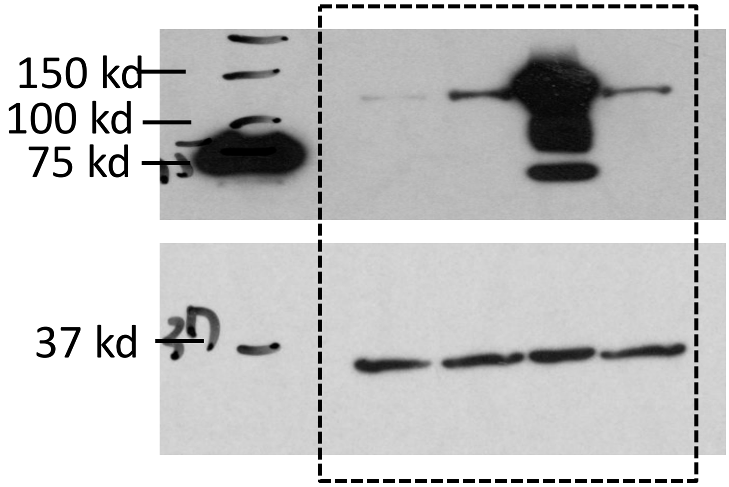

Fig. 5e

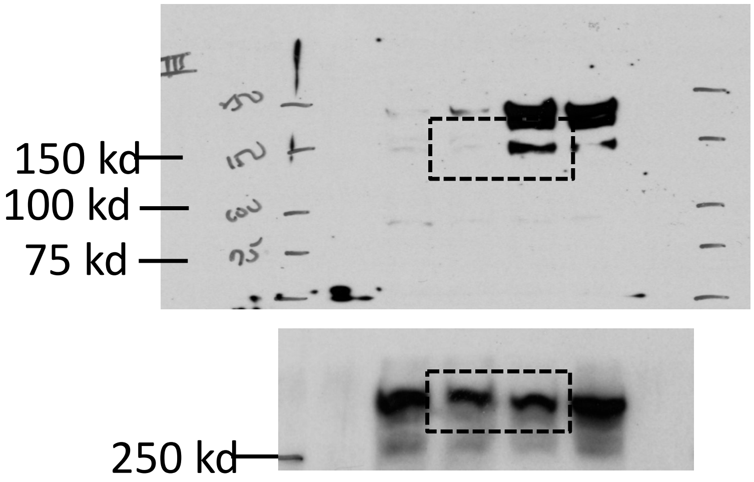

Fig. 6a

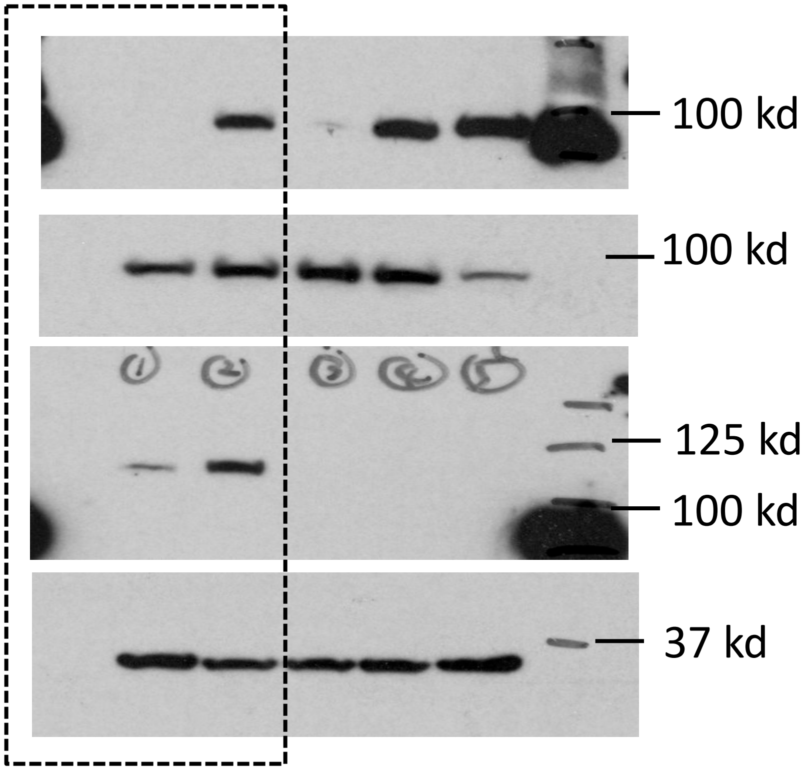

Fig. 6b (left panel)

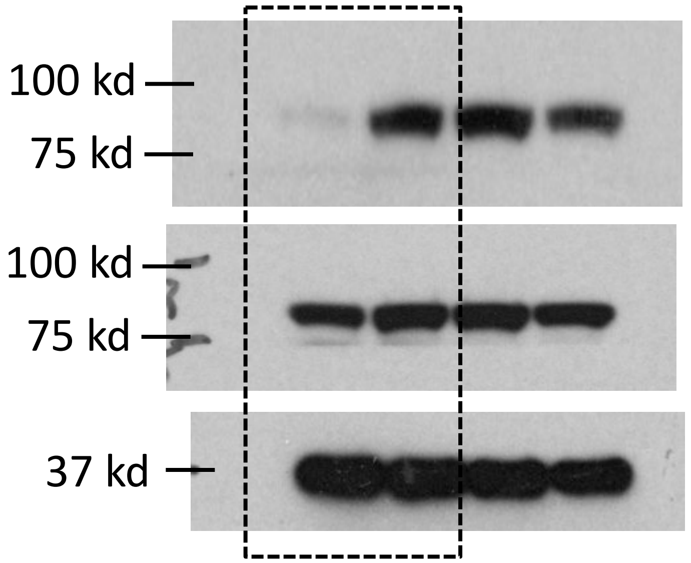

Fig. 6b (right panel)

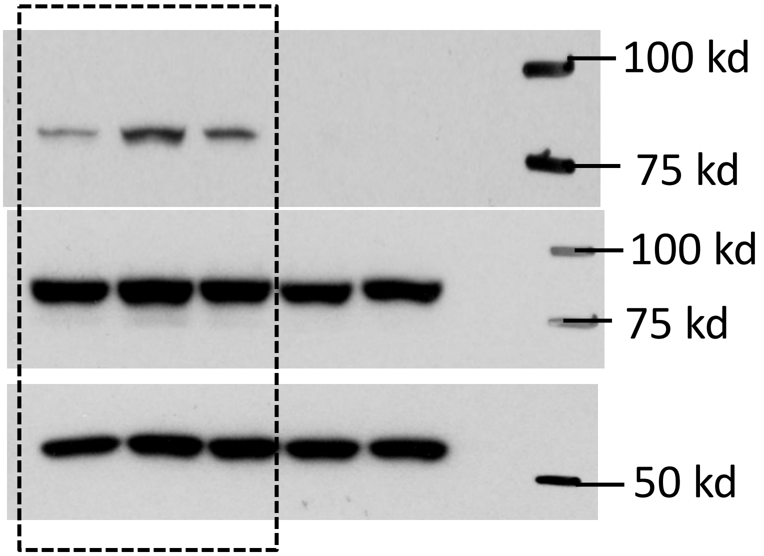

Fig. 6c (left panel)

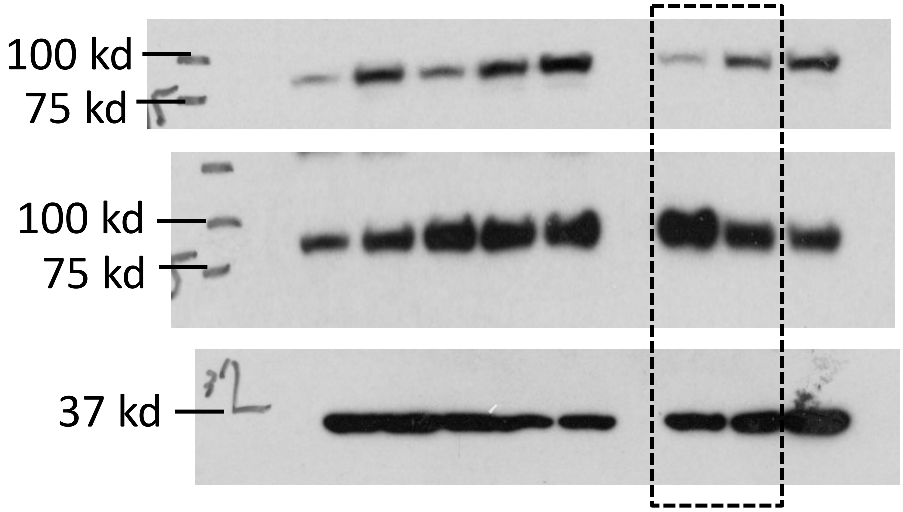

Fig. 6c (right panel)

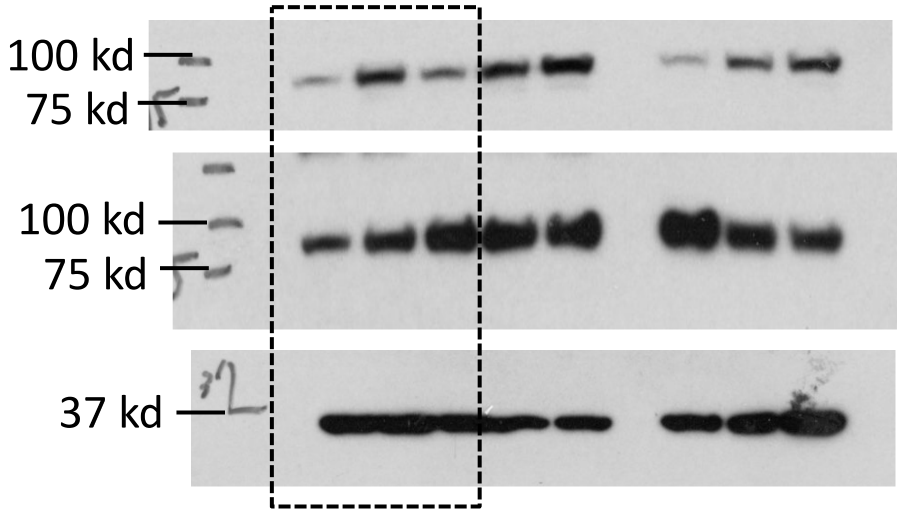

Fig. 6e (left panel)

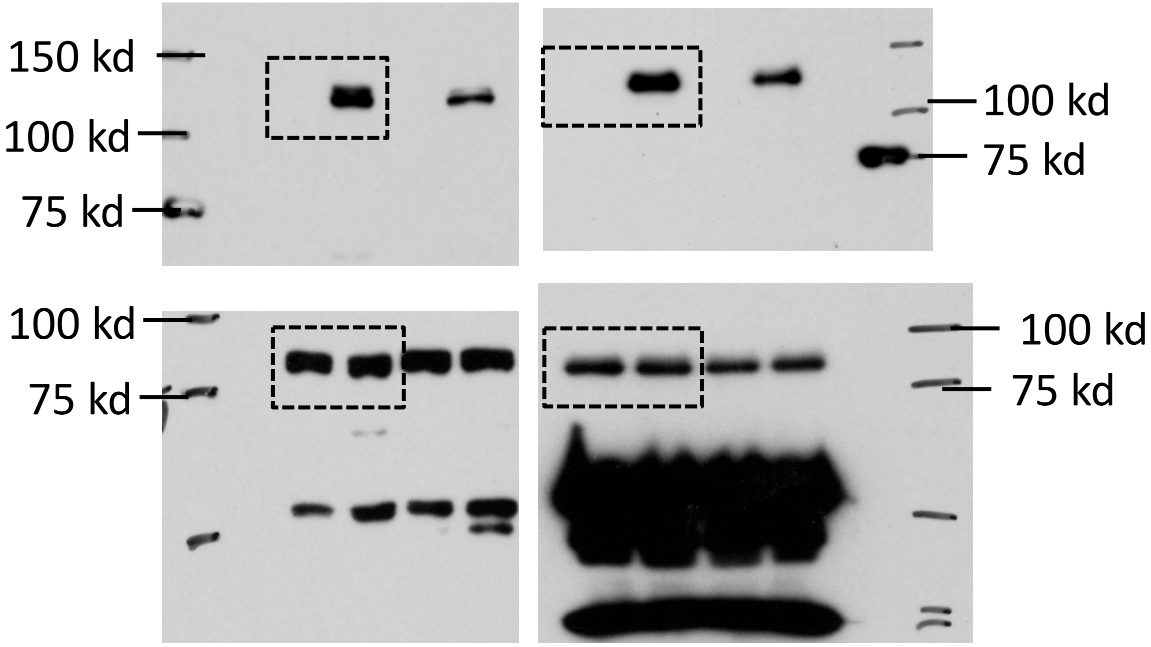

Fig. 6e (right panel)

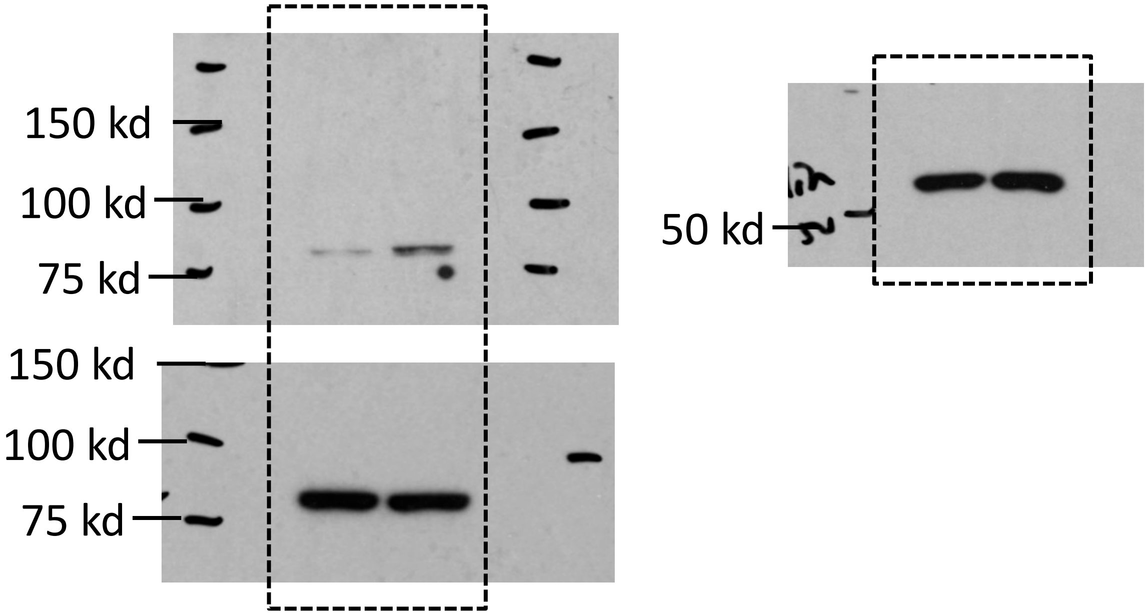

Fig. 6f

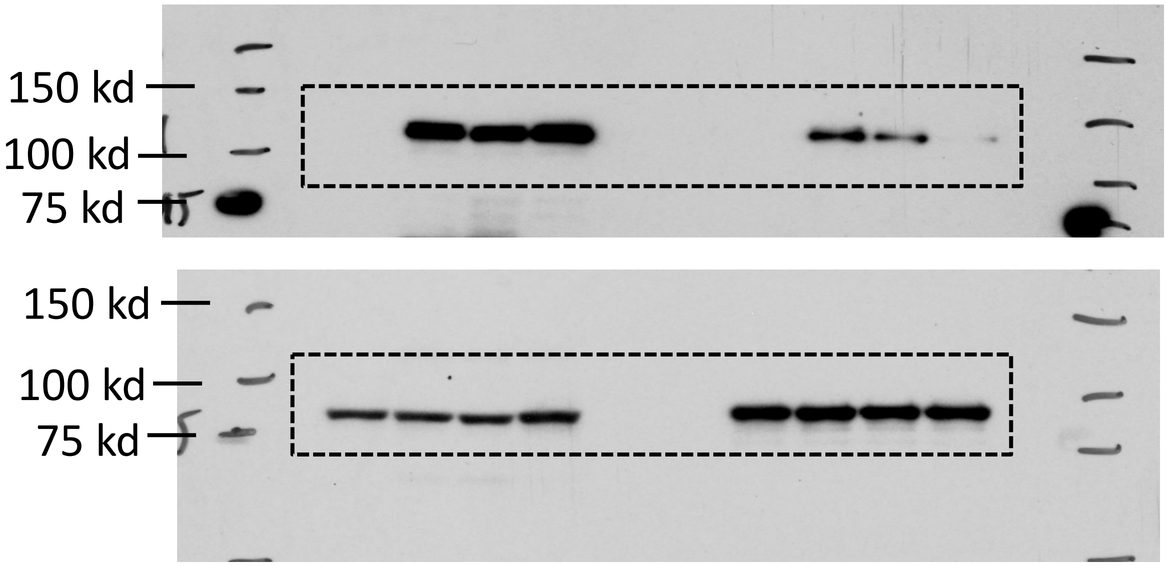
150 kd
100 kd
75 kd
150 kd
100 kd
75 kd

Fig. 6g

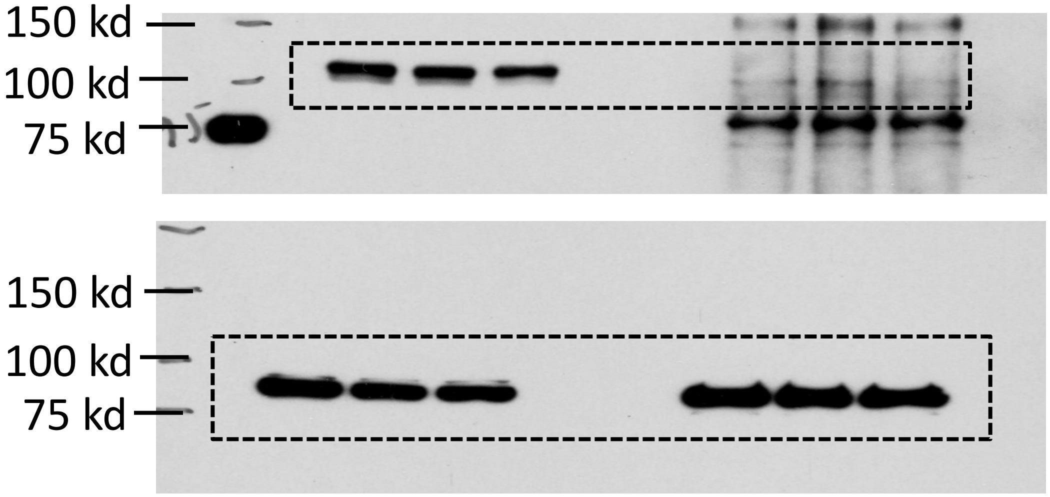
150 kd
100 kd
75 kd
150 kd
100 kd
75 kd

Fig. 6h

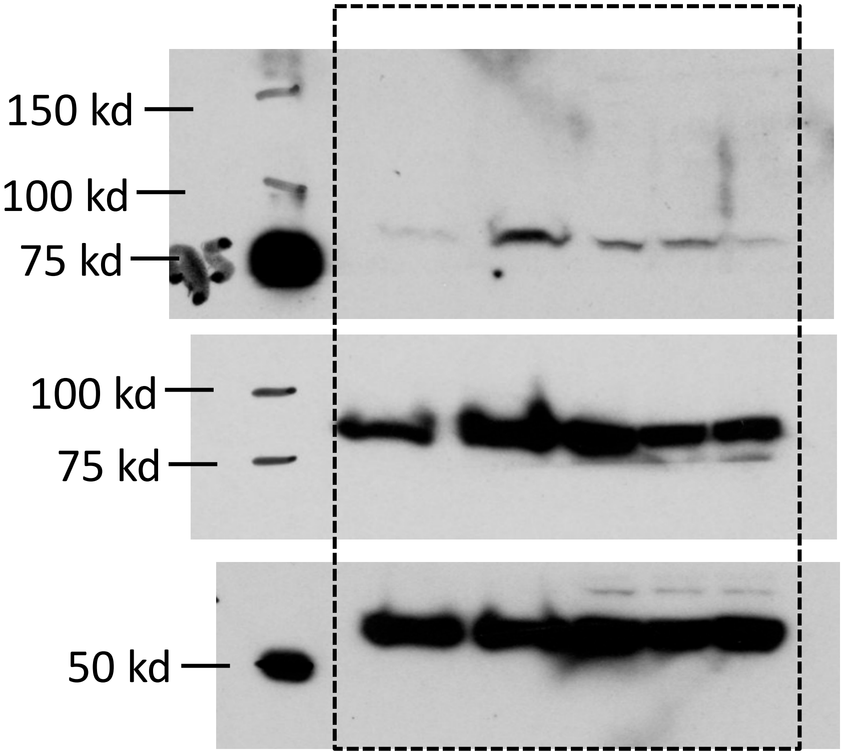
150 kd
100 kd
75 kd
100 kd
75 kd
50 kd

Fig 6i

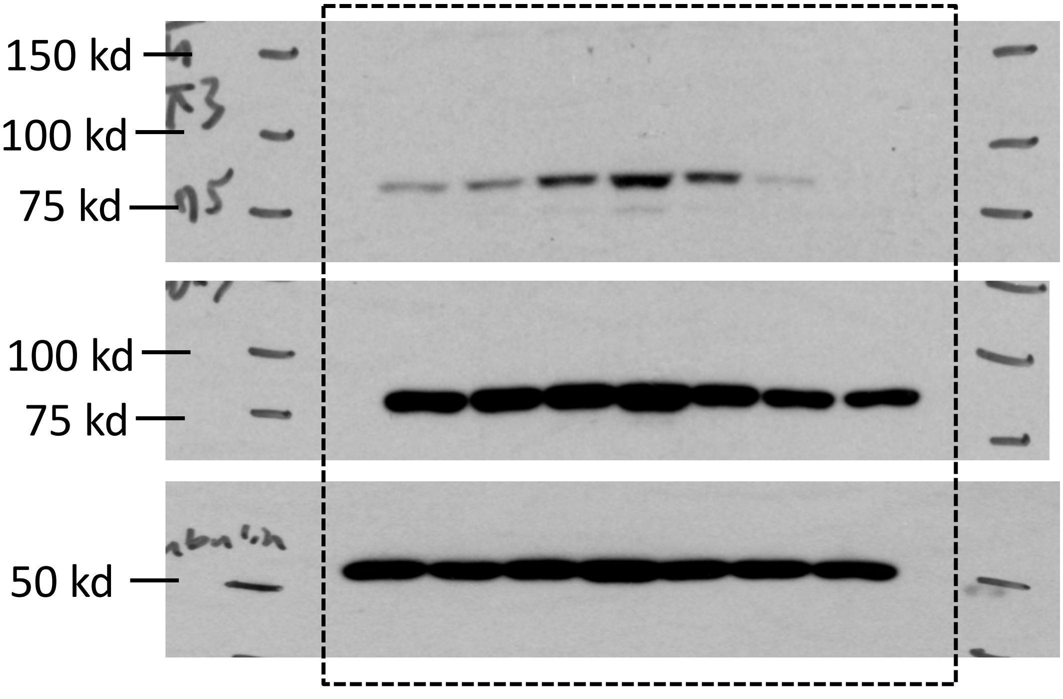
150 kd
100 kd
75 kd
100 kd
75 kd
50 kd

Fig. 6j

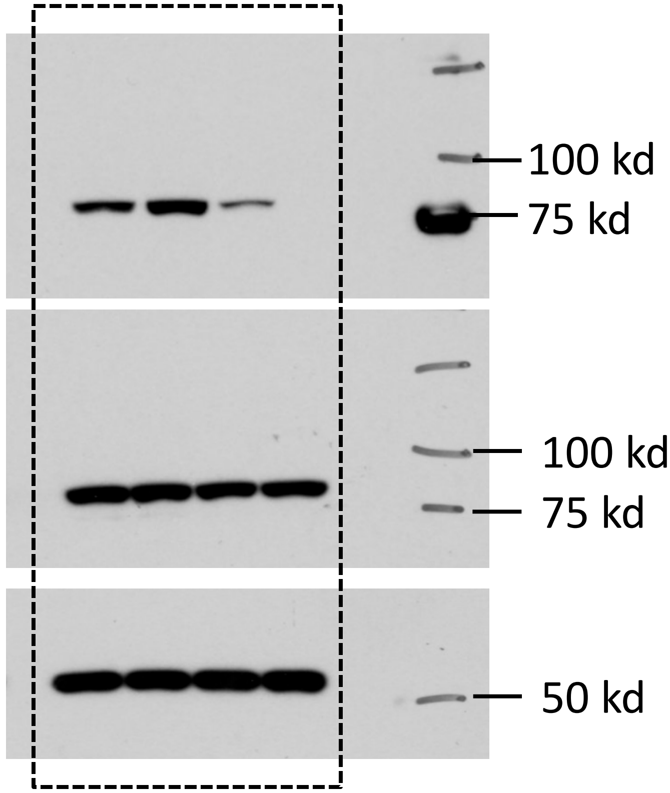

Fig. 6k

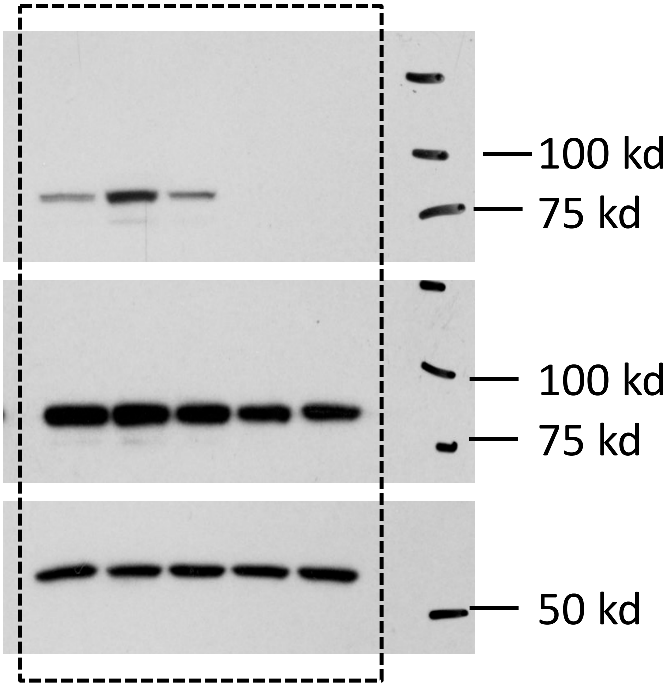

Supplement: Supplementary file 3 — Source Data [file 41467_2019_9878_MOESM3_ESM.zip › Source Data.pdf]
